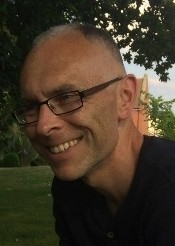# Thiazolidinediones: An In–Depth Study of Their Synthesis and Application to Medicinal Chemistry in the Treatment of Diabetes Mellitus

**DOI:** 10.1002/cmdc.202100177

**Published:** 2021-05-04

**Authors:** Nathan Long, Adam Le Gresley, Stephen P. Wren

**Affiliations:** ^1^ Department of Chemical & Pharmaceutical Sciences Faculty of Science Engineering & Computing Kingston University London Penrhyn Road Surrey KT1 2EE UK

**Keywords:** Thiazolidinediones, Diabetes Mellitus, PPARγ, PTP1B, ALR2

## Abstract

2,4‐Thiazolidinedione (TZD) is a privileged and highly utilised scaffold for the development of pharmaceutically active compounds. This sulfur‐containing heterocycle is a versatile pharmacophore that confers a diverse range of pharmacological activities. TZD has been shown to exhibit biological action towards a vast range of targets interesting to medicinal chemists. In this review, we attempt to provide insight into both the historical conventional and the use of novel methodologies to synthesise the TZD core framework. Further to this, synthetic procedures utilised to substitute the TZD molecule at the activated methylene C_5_ and N_3_ position are reviewed. Finally, research into developing clinical agents, which act as modulators of peroxisome proliferator‐activated receptors gamma (PPARγ), protein tyrosine phosphatase 1B (PTP1B) and aldose reductase 2 (ALR2), are discussed. These are the three most targeted receptors for the treatment of diabetes mellitus (DM).

## Graphical abstract

**A key scaffold**: 2,4‐Thiazolidinedione is a widely recognised structural motif present in many therapeutic agents used for the treatment of diabetes mellitus. This review summarises some of the methods used to prepare biologically active members of this class and discusses the pharmacological potency of these compounds.

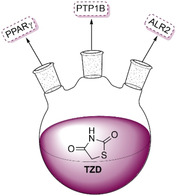

## 1 Introduction to TZD

Heterocyclic systems are recognised to be of great importance due to their proven utility within the field of medicinal chemistry.^[1]^ It has been estimated that more than 85 % of all chemical entities which evoke a biological reaction contain at least one heterocycle.^[2]^ The incorporation of heterocycles into drug molecules allows for organic chemists to modulate pharmacokinetic and pharmacodynamic properties, by altering such parameters as lipophilicity, polarity, hydrogen bonding ability as well as toxicological profiles.^[2]^ It is, therefore, unsurprising that organic chemists have become highly familiar with heterocycles featuring various ring sizes.

**Figure 1:**
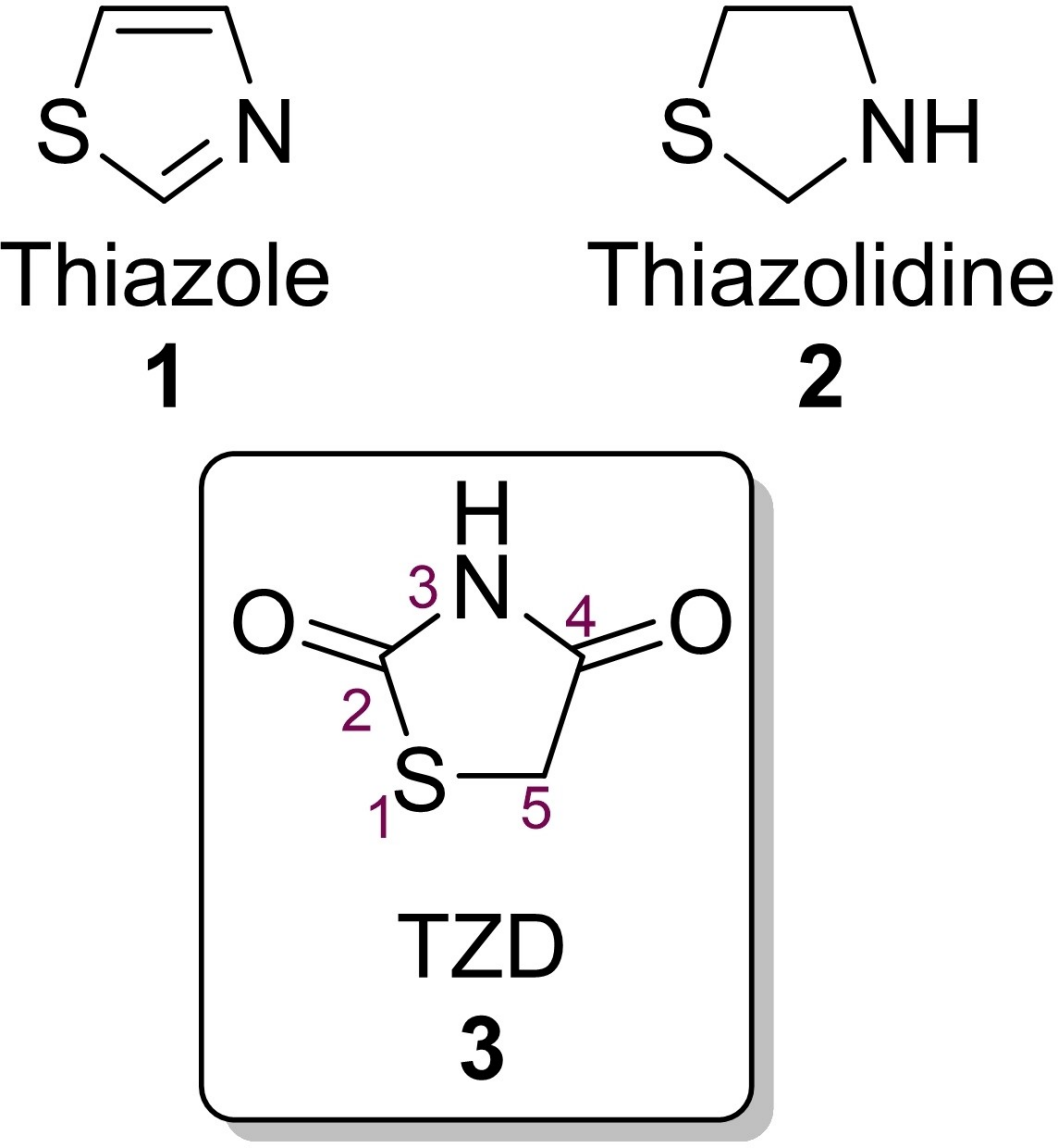
Structures of thiazole, thiazolidine and TZD.

One such five‐membered ring heterocycle is a thiazole (**1**), its non‐aromatic analogue being thiazolidine (**2**). When **2** is decorated further with two carbonyl groups at position 2 and 4, the ring system is termed 2,4‐thiazolidinedione (**3**) (TZD), which is the focus of this review (Figure 1). TZD exists as a white crystalline solid with a melting point of 123–125 °C and is bench stable when kept below 30 °C.^[3]^ In terms of solubility, TZD is only sparingly soluble in a variety of common organic solvents including water, MeOH, EtOH, DMSO and Et_2_O.^[4]^

**Figure 2:**
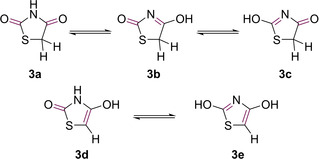
Tautomeric structures of TZD.

Due to the presence of two carbonyl groups and an α‐hydrogen, TZD has the ability to exist as a series of tautomers (see **3 a**–**e**, Figure 2).^[3]^

Aside from its use within the sphere of organic and medicinal chemistry, TZD acts as an inhibitor for the corrosion of steels in acidic environments and is reported as a ‘brightener’ in the electroplating industry.^[5]^

### 1.1 TZD core synthesis

**Scheme 1:**
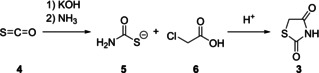
Initially used methodology to generate TZD using carbonyl sulphide.

Synthetic methodologies to yield the TZD core were first reported in the 1923 work by Kallenberg.^[6]^ Kallenberg's method reacts carbonyl sulphide (**4**) with ammonia, in the presence of KOH, to generate *in situ* the corresponding alkyl thioncarbamate (**5**), which, in turn, reacts with an α‐halogenated carboxylic acid (**6**). The thiocarbamate produced is then cyclised in acidic conditions, to yield the desired TZD (**3**, Scheme 1).^[6]^

**Scheme 2:**
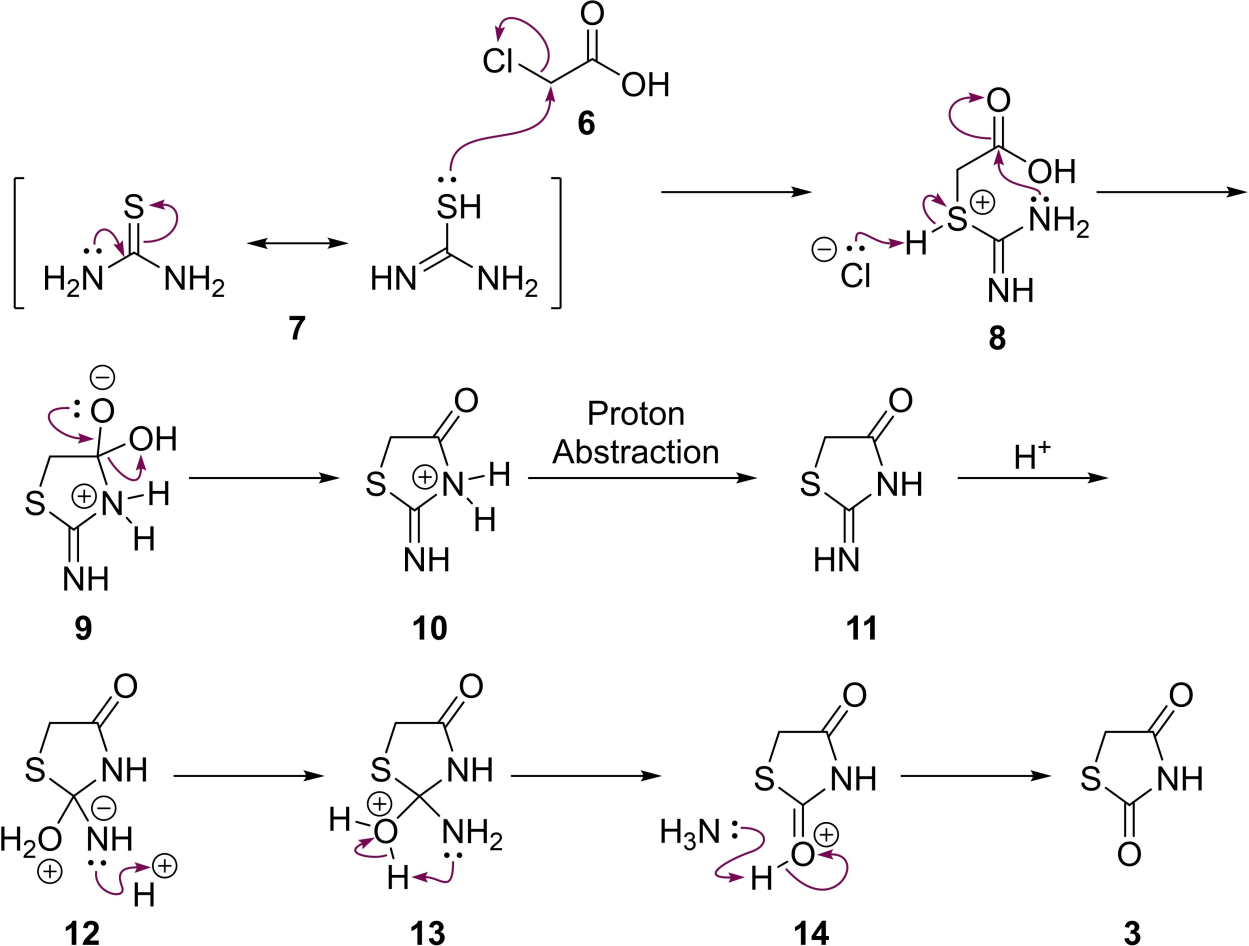
A mechanistic view of the TZD synthesis using thiourea (**7**) and α‐chloroacetic acid (**6**).

Using more recent methodology, TZD is often synthesised by refluxing α‐chloroacetic acid (**6**) with thiourea (**7**), utilising water as a solvent for a prolonged period of time (∼12 h).^[7]^ The reaction mechanism for this process was proposed by Liberman *et al*.^[8]^ and can be seen below (Scheme 2).^[7]^ Here, an initial attack on chloroacetic acid by the thiourea sulfur atom occurs. This S_N_2 type reaction and generation of HCl takes place before a subsequent, second, nucleophilic substitution reaction caused by the attack of the amine onto the carboxylic carbon (releasing water). The final steps of this reaction rely on the generation of the 2‐imino‐4‐thiazolidinone intermediate species (**11**), which is hydrolysed at position 2 by the *in situ* generation of HCl. Such hydrolysis results in the eventual release of ammonia as a gas and yields TZD (**3**).^[8]^

**Scheme 3:**
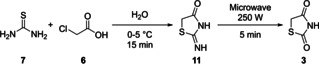
Microwave‐induced synthesis of TZD using thiourea (**7**) and α‐chloroacetic acid (**6**).

The above and previously described methodology requires prolonged heating at significantly elevated temperatures (100–110 °C). In order to overcome such issues, Kumar and colleagues in 2006, evaluated the use of microwave‐induced synthesis to yield **3**. In a push to develop greener synthetic methodologies, it is not surprising that solid phase, solvent‐free reactions promoted/initiated *via* microwave irradiation have seen considerable attention in the last 20 years.^[9]^ The method proposed by Kumar can be completed in a total of two synthetic steps in less than 0.5 h. Both chloroacetic acid (**6**) and thiourea (**7**) were suspended in water and stirred under ice‐cold conditions for approximately 15 min, to precipitate out the previously discussed 2‐imino‐4‐thiazolidinone (**11**).^[10]^ Compound **11** was then subjected to microwave initiation at 250 W for a period of 5 min (Scheme 3). The desired TZD was isolated following cooling and vacuum filtration in 83 % yield and without the need for further purification. Although the yield was the same as the conventional method, the reduction in reaction time and temperature certainly presented some synthetic advantages.^[10]^

**Figure 3:**
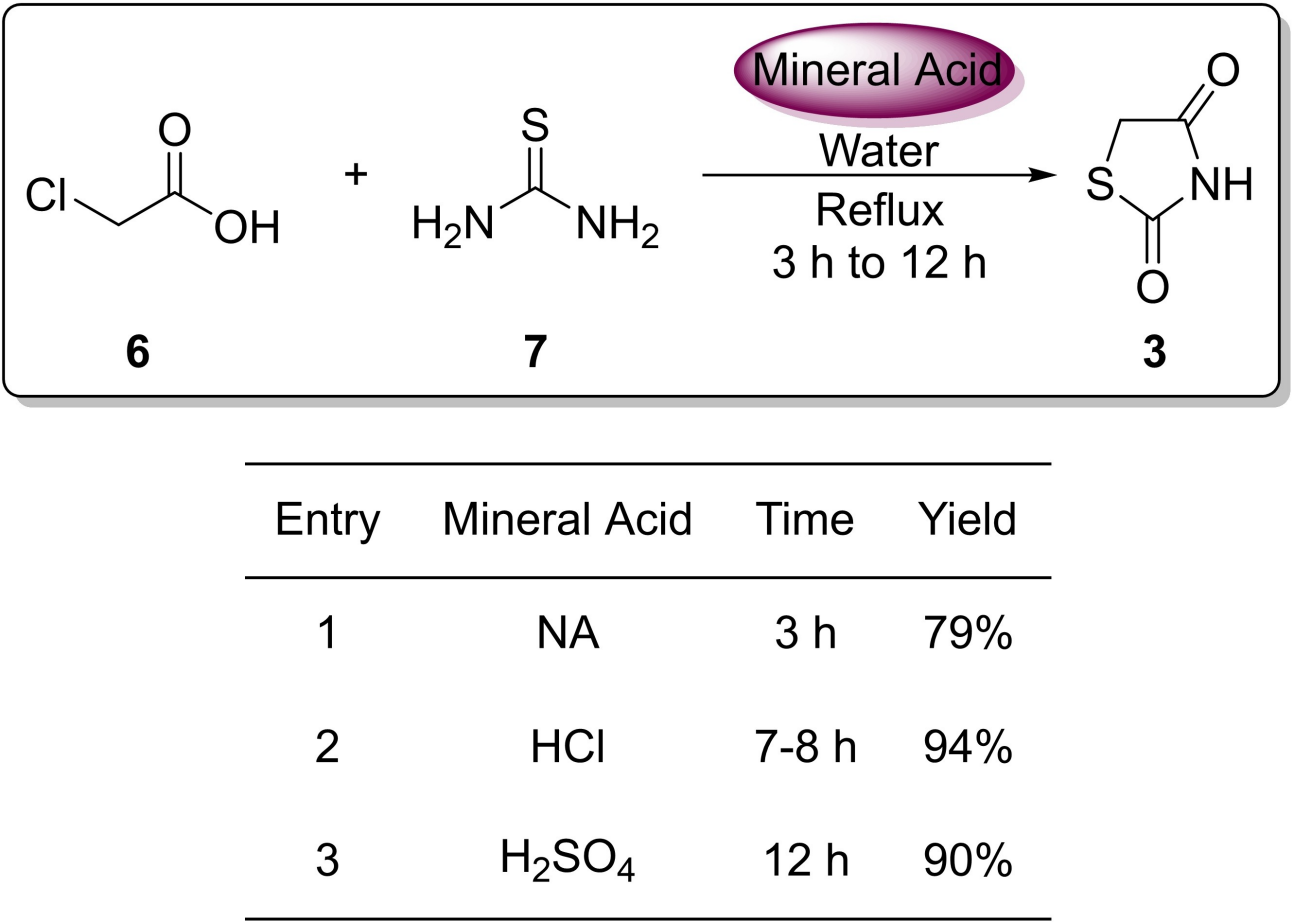
Investigation into the effect of different acids on the yield of TZD.

It should be noted that conducting the reaction utilising HCl as the acid and heating for a 7–8 hour period provided the highest yield of **3** at 94 % (Figure 3).[^11^, ^12^]

**Scheme 4:**
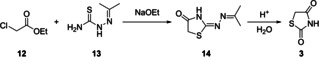
Synthesis of TZD utilising ethyl chloroacetate (**12**) and thiosemicarbazone (**13**).

A third common synthetic protocol involves the reaction of ethyl chloroacetate (**12**) with thiosemicarbazone (**13**), which in the presence of NaOEt, generates 2‐hydrazino‐4‐thiazolidinone (**14**); this, in turn, can be refluxed in dilute hydrochloric acid to give the desired TZD (**3**) (Scheme 4).^[13]^

**Scheme 5:**
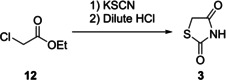
Synthesis of TZD using ethyl chloroacetate and potassium thiocyanate.

A fourth commonly used method to generate the TZD core also utilises compound **12**. Here, acidification of the product obtained from the reaction of **12** with potassium thiocyanate yields **3** (Scheme 5). It should be noted that during this process significant care must be taken due to the liberation of toxic HCN gas as a by‐product.^[14]^

Though a series of methodologies have been presented to generate **3** as a core framework the majority of research has been laid into developing robust, high yielding and simple substitution reactions. While novel methodologies have been illustrated above, the most widely used synthetic pathway to produce the TZD core remains as that illustrated in Scheme 2 and Figure 3. This is because reagents utilised are available in large quantities from commercial sources and do not require the handling or liberation of severely toxic by‐products (as seen in the case of Scheme 5). Despite the efficient synthesis of **3** with aid of a microwave reactor the same yield was achieved.

## 2 Substitution Reactions

**Scheme 6:**
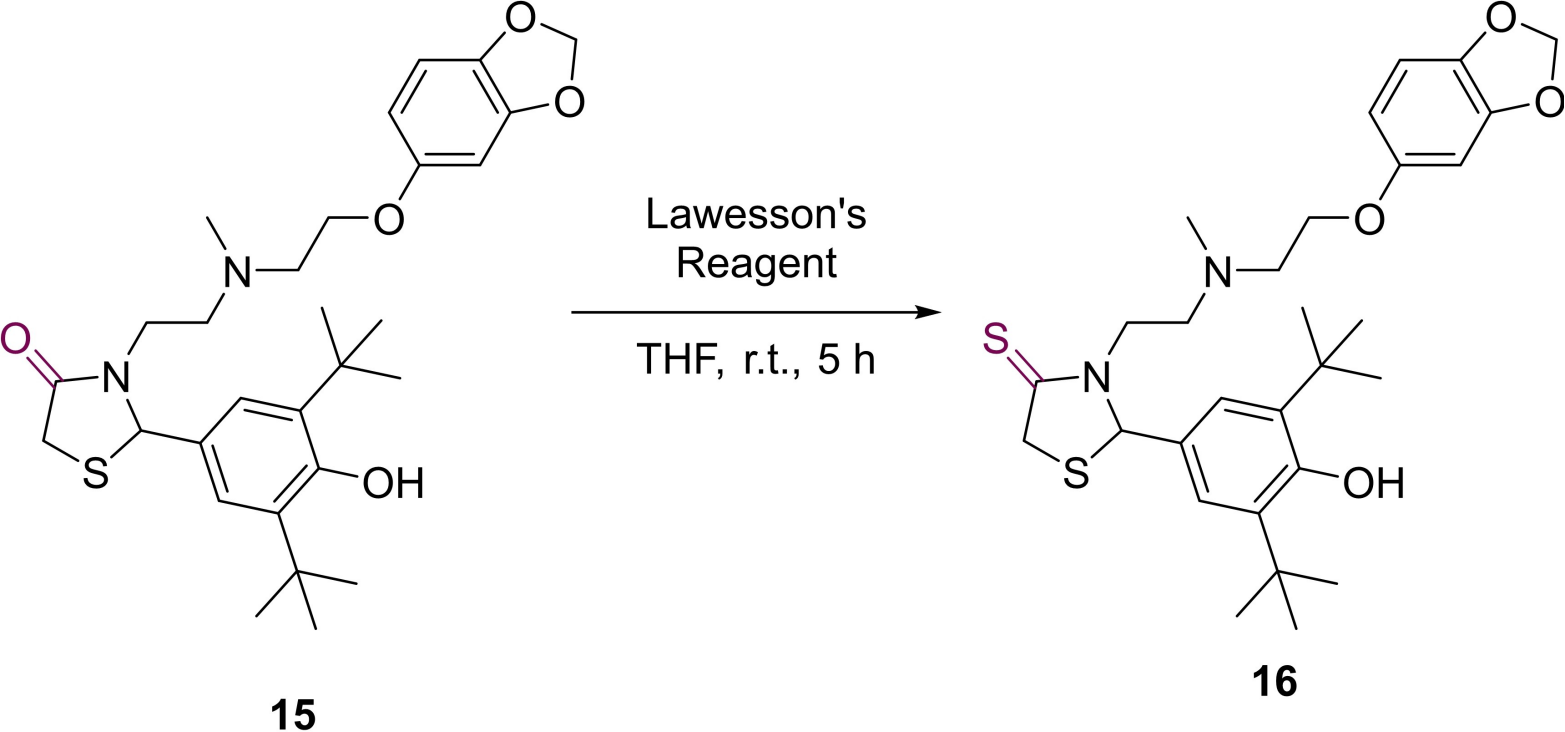
Generation of thiocarbonyl derivatives with aid of Lawesson's Reagent.

The large number of reported substitutions on the core TZD frame can result in the alteration of molecular properties, the generation of novel molecules and potentially bioactive candidates. Substitutions at the nitrogen position N_3_, the C_2_ carbonyl and the methylene group are known.^[15]^ The carbonyl group present at C_4_ is considered highly unreactive.^[15]^ The only reported reactivity at this position was displayed in 1999 by Kato *et al*. When **15**, a derivative of **3**, was treatted with Lawesson's reagent in THF to afford the corresponding thiocarbonyl compound (**16**) (Scheme 6).^[16]^

### 2.1 NH substitution

**Scheme 7:**
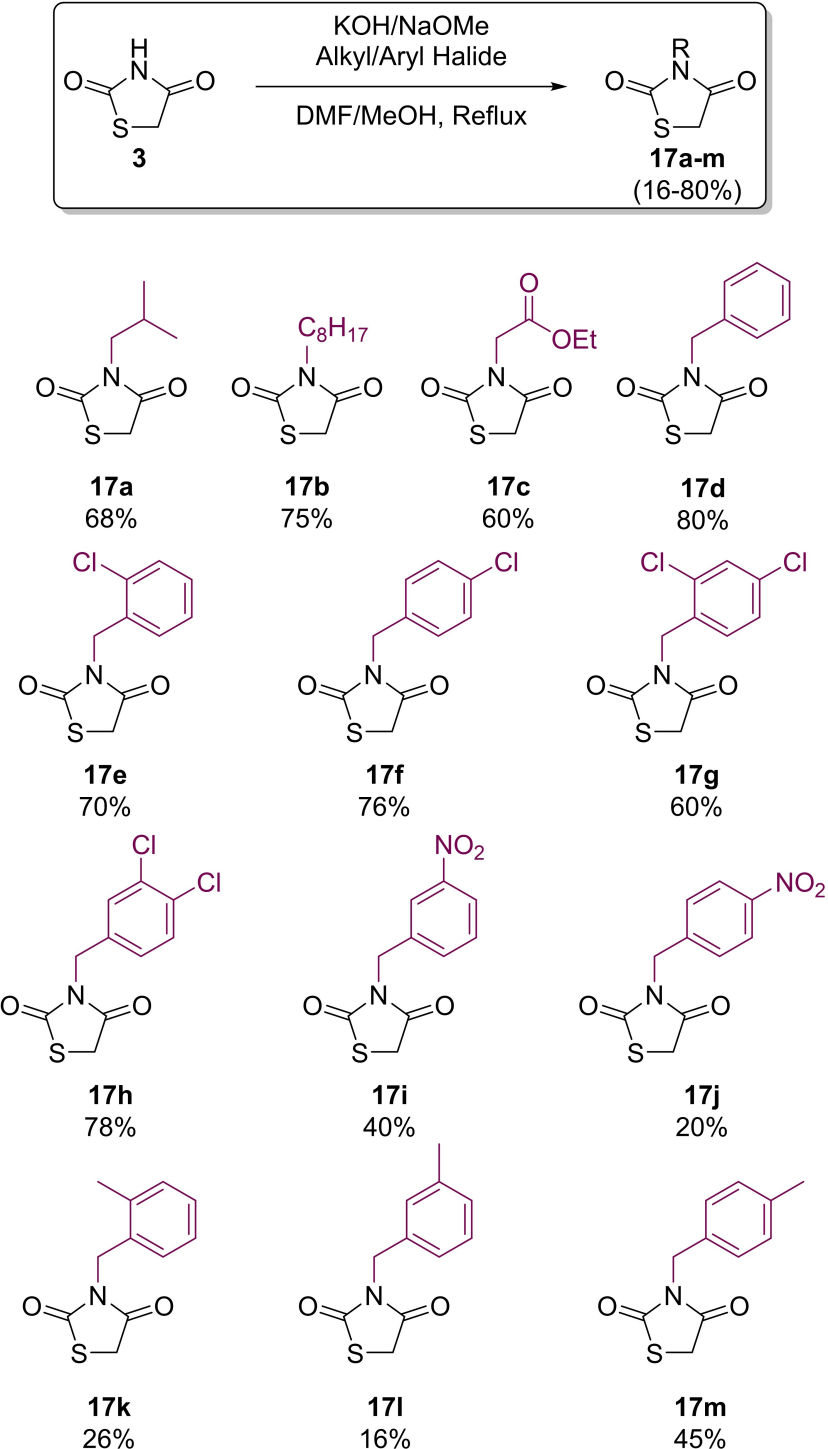
Generation of *N*‐substituted TZDs following reported procedure by Bradsher and Lo.[^17^, ^18^]

The primary methodology to introduce substituents onto the nitrogen atom involves deprotonation with an appropriate base, followed by substitution with either alkyl or benzyl halides. The first reported generation of such compounds dates back to the mid 1950’s in work by Lo and Bradsher.[^17^, ^18^] Their protocols used either NaOMe (Bradsher) or KOH (Lo) as the base. When NaOMe is utilised, Bradsher opted to conduct the reaction in hot MeOH whereas this was exchanged for DMF in alkylation reactions performed by Lo (Scheme 7).[^17^, ^18^] Over time, with significant research directed towards conducting *N*‐alkylation of TZD frameworks, a vast range of different bases have been screened. It is now readily accepted that other suitable bases include potassium carbonate, tetrabutylammonium iodide^[19]^, NEt_3_ ^[20]^ or even sodium hydride.^[21]^ It has also been determined that suitable solvents include acetone.

**Scheme 8:**
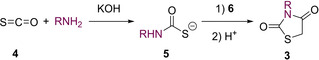
Generation of *N*‐substituted TZD derivatives utilising carbonyl sulphide (**4**) and a primary amine.

Another less commonly used methodology for the generation of 3‐substituted TZDs, relates back to one of the original procedures used for the generation of the TZD core (**3**). By substituting ammonia with a primary amine (Scheme 8), the substituent group present is shown to persist through the entire process and results in the desired *N*‐substituted derivatives.^[22]^

**Scheme 9:**
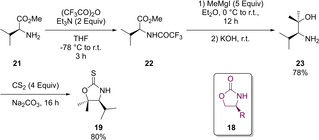
Generation of a chiral auxilary resembling TZD.

A final and more novel approach can be seen in a 2005 paper published by Mendoza that discloses access to *N*‐substituted TZD structures derived from oxazolidinethiones.^[23]^ Chiral auxiliaries have been used to construct bioactive compounds.^[24]^ The chiral auxiliary used in Mendoza‘s work can be seen as a derivative of the renowned Evans auxiliary (**18**), with the only difference being a replacement of the carbonyl function to a thione (**19**). Preparation of **18** initially involves protection of *S*‐valine methyl ester (**21**) amino group using trifluoroacetic anhydride and NEt_3_ to generate *N*‐trifluoroacetyl *S*‐valine methyl ester (**22**). Subsequent addition of methyl magnesium iodide followed by ester hydrolysis, in the presence of aqueous potassium hydroxide, gives β‐amino alcohol **23** in 78 % yield overall. The final synthetic step which induces cyclisation to form **19** (80 %) also installed the thione group (Scheme 9).^[23]^

**Scheme 10:**
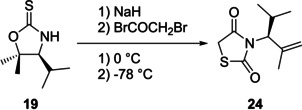
Synthesis of an *N*‐substituted TZD from **19**.

With the chiral auxiliary in hand, the Mendoza group managed to induce a shift of the isopropyl group occupying the C_4_ position to the nitrogen and transform the oxazolidine‐2‐thione core moiety into the one featured in TZD. The procedure involved treating **19** with NaH at 0 °C in dichloromethane, prior to the dropwise addition of bromoacetyl bromide at −78 °C. The product (**24**), was generated as an oil in 67 % yield (Scheme 10).^[25]^

### 2.2 Methylene substitution

**Figure 4:**
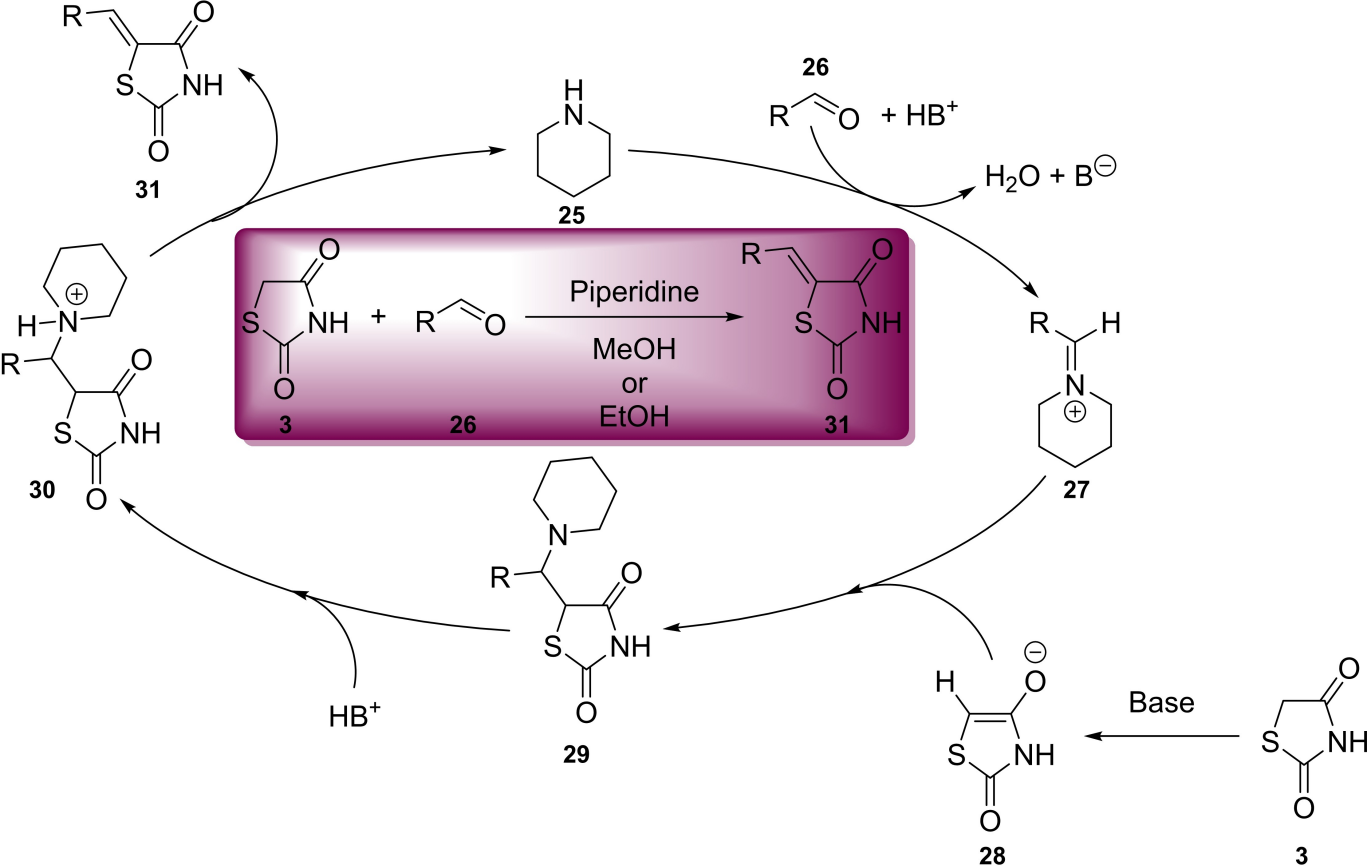
KC of TZD and an appropriate aldehyde utilising piperidine as a base and catalyst.

Further key functionalisation of the TZD framework can be achieved through substitution at the methylene C_5_ position. The most widely used methodology involves a Knoevenagel condensation (KC), which relies upon the addition of an aldehyde to an activated methylene unit followed by a dehydration reaction to generate a new olefin.^[26]^ The reaction is often conducted in the presence of a base and these often include primary and secondary amines.^[27]^ The base causes an initial removal of the acidic proton before its nucleophilic attack onto the carbonyl (**28**, Figure 4). Following this, the conjugate base induces an elimination reaction with the production of water. In the pursuit of developing a series of 5‐(substituted benzylidene)‐TZD derivatives to act as tyrosine kinase inhibitors, Ha and co‐workers discovered that a catalytic amount of piperidine is optimal for this type of chemistry.^[28]^ A proposed catalytic cycle involving the use of piperidine is shown below in Figure 4. The reaction can tolerate bases such as NaOH^[29]^, MeNH_2_, morpholine, K_2_CO_3_ and aqueous ammonia. Typically, the condensation is conducted in standard organic solvents such as alcohols, DMF, DMSO or DCM.^[30]^

Though robust, the standard KC conditions involve prolonged heating (usually for up to 12 h) and complicated azeotropic removal of H_2_O with the aid of a Dean‐Stark apparatus and, as such, results in a time‐consuming and tedious reaction. Fortunately, more convenient methods have been developed over the last twenty years. In 2006, a solvent‐free synthetic protocol was discovered. In this work it was found that combining both initial precursors along with piperidine (to act as a base and catalyst), activated silica gel and acetic acid within a 900 W microwave reactor, gave reaction times of ∼7 minutes.^[10]^ Kumar found that not only did the reaction time decrease but the yield of all 23 substrates tested also increased. The removal of the activated silica and/or the AcOH decreased the yield.^[10]^

**Scheme 11:**
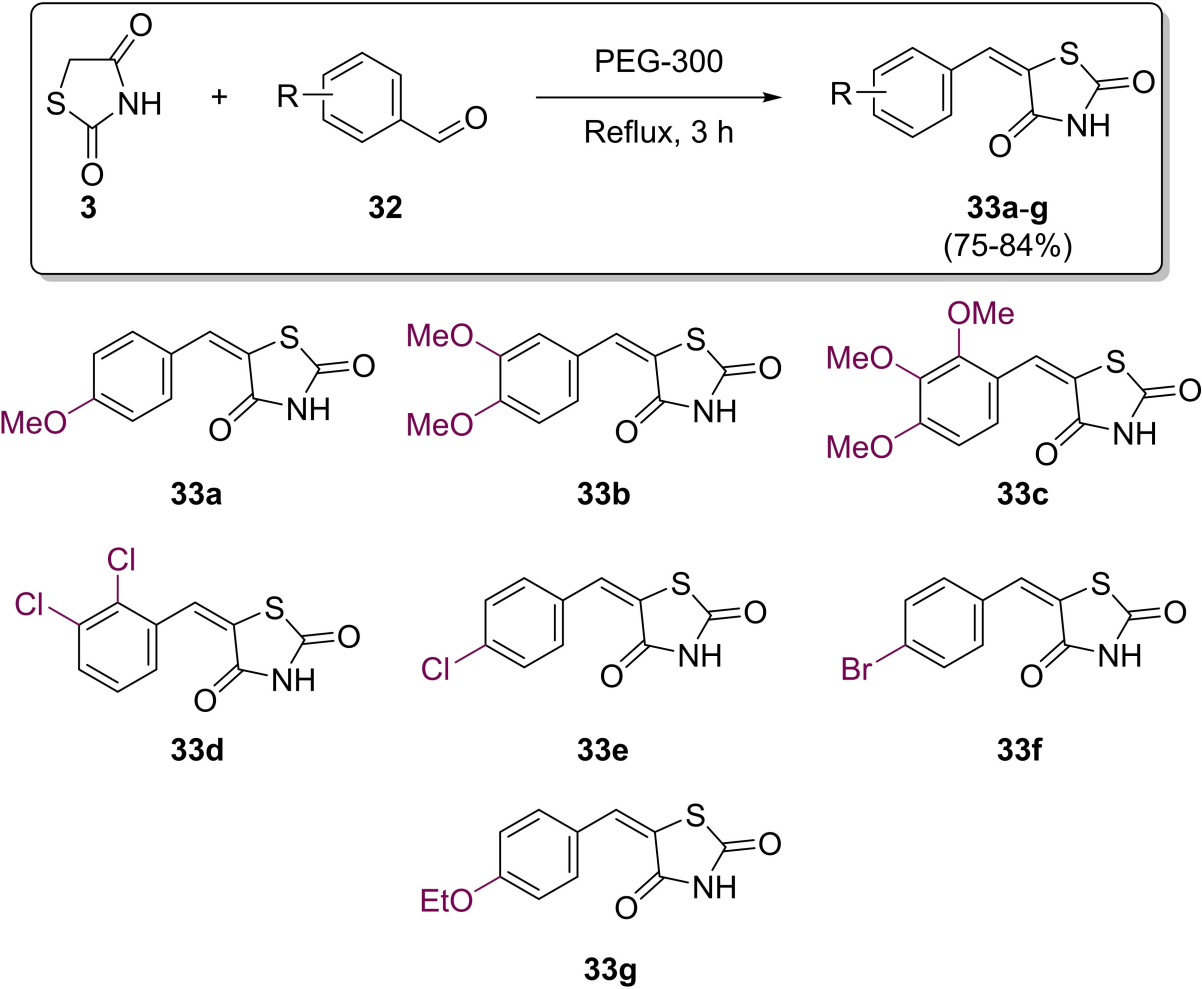
Methylene substition to yield 5‐benzylidene derivatives utilising PEG‐300.

In work aimed at generation of greener methodology, Mahalle *et al*, avoided the use of hazardous catalysts/solvents by using polyethylene glycol (PEG‐300) instead. The group found that refluxing both the aldehyde and TZD in PEG for around 3 h resulted in significantly high yields of up to 80 % (Scheme 11).^[30]^

**Scheme 12:**
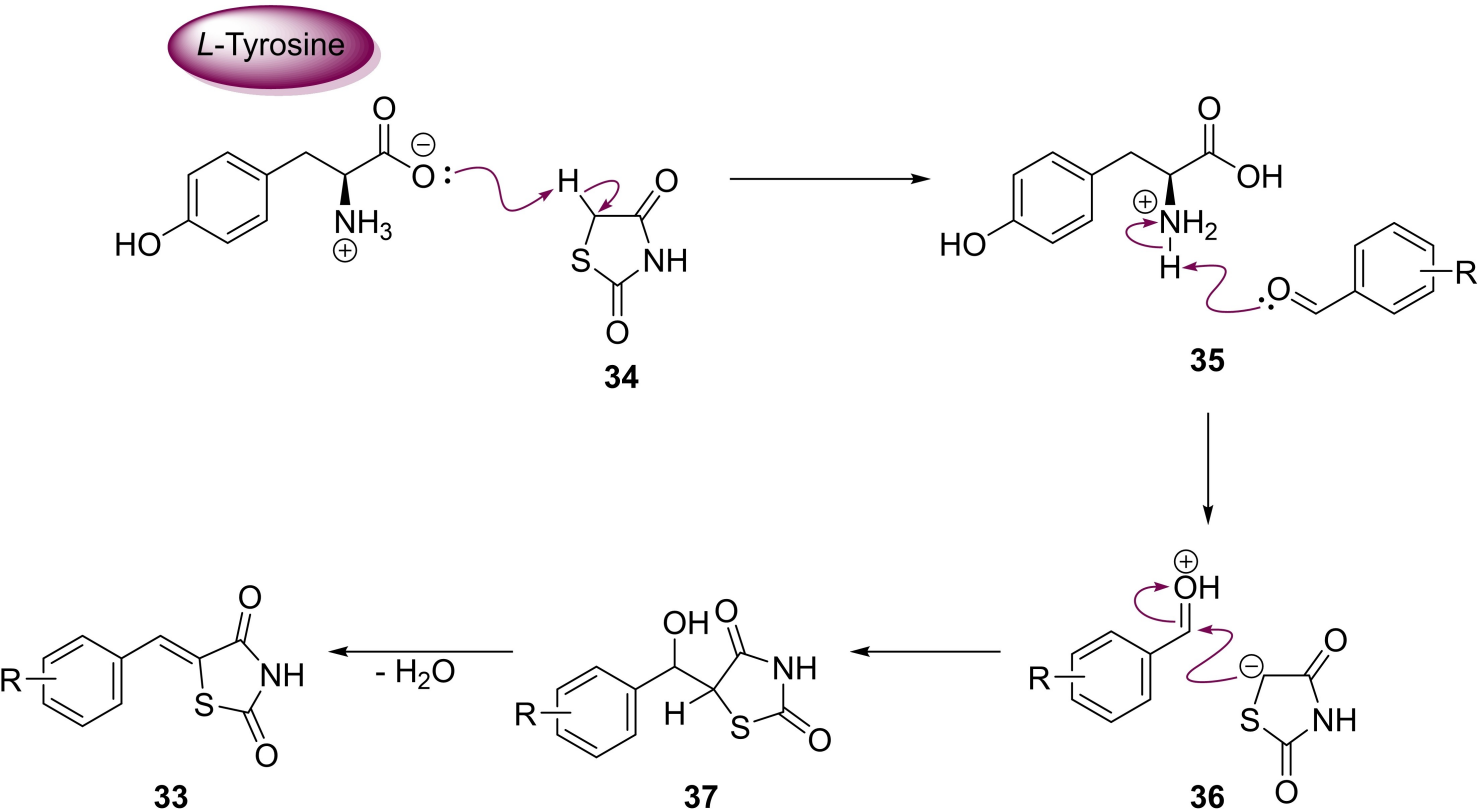
Methylene substitution to yield 5‐benzylidene derivatives utilising L‐tyrosine.

In 2012, Thirupathi and co‐workers looked to the biochemical world in search of a cheap, non‐toxic, and readily available catalyst to be used in a modified Knoevenagel reaction. The catalyst which proved to be most promising was *L*‐tyrosine. They developed a highly efficient protocol, conducted at ambient temperature, utilising water as a solvent, with a fast work‐up and purification procedure and rapid reaction times. The proposed reaction mechanism for the adapted procedure can be found below (Scheme 12). In their research to generate a series of 11 analogues (**33**), they found that aryl aldehydes substituted with electron‐withdrawing groups (EWG) reacted faster than those possessing electron‐donating groups (EDG). It was also found that *L*‐tyrosine is essential for reactivity as no product formation occurred when it was absent.^[31]^

The initial mechanistic step features *L*‐tyrosine in its zwitterionic form abstracting a proton from the activated methylene of TZD (**34**). Then, the carbonyl is protonated to the corresponding oxonium cation (**35**) before it is attacked by the highly nucleophilic deprotonated TZD core (**36**). The final step once again involves a dehydration reaction in which the desired 5‐arylidene compounds are formed (**33**) (Scheme 12).

**Scheme 13:**
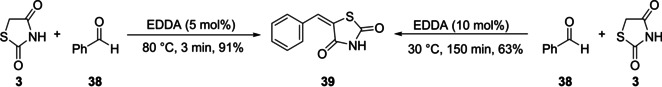
Synthesis of 5‐benzylidene derivative via KC using EDDA catalyst.

In the same year, a second modified Knoevenagel protocol was developed by Zhou and co‐workers at the South‐Central University of Nationalities, Wuhan.^[32]^ They utilised ethylenediamine diacetate (EDDA), a readily available and cheap Brønsted acid‐base combined salt catalyst, which has generated considerable interest in recent times.[^33^, ^34^] The coupling of benzaldehyde (**38**) with TZD at room temperature, in a solvent‐free environment with catalyst loading set to 10 mol% produced the desired 5‐benzylidene (**39**) in a 63 % yield after a reaction time of 150 min (Scheme 13). The optimal reaction conditions consisted of conducting the reaction at 80 °C with 5 mol% catalyst loading, giving rise to a 91 % yield of **39** (Scheme 13).^[32]^

**Scheme 14:**
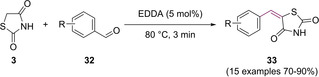
Methylene substitution to yield a series of 5‐benzylidene analogues using EDDA catalyst.

No product was yielded in the absence of EDDA. From a green synthesis perspective, Zhou and colleagues also looked at the possibility of recycling the EDDA catalyst.^[32]^ The filtrate containing the EDDA contaminated water was concentrated *in vacuo* and used directly in a subsequent reaction. Results from this study showed that the recovered EDDA could be used in at least four reactions before considerable reduction in catalyst activity was observed.^[32]^ The group successfully managed to synthesise a series of benzylidene structures (**33**) in significantly high yields ranging from 70–91 % (Scheme 14).^[32]^

Over the past few decades, the use of ultrasound to promote chemical reactions has seen an increase in popularity.[^35^, ^36^] Sonochemical techniques can be seen as a milder alternative to conventional reaction heating and has demonstrated the ability to provide higher yields with shorter reaction time all without the need for inert reaction conditions.^[36]^

**Scheme 15:**
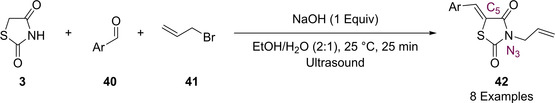
Generation of disubstituted TZD derivatives through sonification.

With these advantages in mind, Bougrin *et al*. devised a unique methodology for the synthesis of novel TZD containing structures substituted at both N_2_ and C_5_ position *via* a one‐pot synthetic protocol. This involved combining TZD, along with the desired aldehyde that would effectively substitute at the C_5_ position, and an alkyl halide (to react at the amine centre). This work is illustrated by the reaction of **3**, **40** and **41** in Scheme 15. The reagents are dissolved in a mixture of EtOH/H_2_O (*v/v*, 2 : 1) and NaOH before sonification for a period of 25 min at 25 °C (Scheme 15). The desired disubstituted product was isolated following acidic precipitation with 4 M HCl and recrystallisation from hot EtOH. To verify the effect of ultrasound irradiation on reaction success, a series of control experiments were conducted where sonification was exchanged for conventional mechanical magnetic stirring. In these cases, the reaction times were extended up to 12 h without full conversion.^[37]^

Although a vast variety of methodologies have been illustrated which effectively substitute the two main positions of the TZD core framework (C_5_ and N_3_), the most commonly used methodologies are those which are first mentioned. For methylene (C_5_) functionalisation, KC condensation employs mild reaction conditions, high functional group tolerance and generally allows high yielding reactions over a short reaction time. While other methodologies presented herein are novel, they often rely upon the use of specialist equipment or extended work‐up procedures. The same can be said for substitution at the N_3_ position which usually is conducted through deprotonation (using an inorganic base such as NaOH) followed by nucleophilic attack on an alkyl/aryl halide.

## 3 Role of TZDs in Medicinal Chemistry

Due to the diverse range of reactions that can be conducted on the TZD core framework it is unsurprising that substituted TZDs exhibit a vast range of pharmacological activities. Since their first reported use in 1954, TZD derivatives have been known to provide antimicrobial, antiviral, antioxidant, anticancer, anti‐inflammatory, anti‐plasmodial and anti‐hyperglycemic effects.^[38]^ Further to this, TZD containing compounds have demonstrated uses as Al‐2 quorum sensing inhibitors, aldose reductase inhibitors,^[39]^ alpha glucoside inhibitors, COX inhibitors,^[40]^ 15‐hydroxyprostaglandin dehydrogenase inhibitors, peptide deformylase inhibitors,^[38]^ PTP1B inhibitors as well as FFAR1 agonists,^[41]^ β_3_ agonists, GPR‐40 agonists^[42]^ and as peroxisome proliferator‐activated receptors (PPAR) modulators.^[38]^ Aside from substituted TZDs conferring pharmacological effects towards a vast range of protein targets, the motif itself is a recognised bioisostere for the carboxylic acid moiety.

### 3.1 TZD as a bioisostere

A known methodology often utilised in the development of marketable drugs is the exchanging of functional groups for bioisosteric motifs. Bioisostere is recognised as an umbrella term which can be divided into two categories; classical and non‐classical.^[43]^ Classical bioisosteres are described as isoelectronic with the unexchanged moiety and exhibit similar biological properties to the parent structure.^[44]^ Non‐classical bioisosteres however can vary widely, e. g. featuring different number of atoms present, different number of hydrogen bond acceptors (HBA) or donors (HBD), but the electrostatic map is often highly similar to the parent molecule.[^45^, ^46^]

Despite the ubiquitous presence of the carboxylic acid group within endogenous substances (including amino acids, triglycerides, prostaglandins), and its appearance in over 450 known marketed drugs worldwide (including NSAID's, antibiotics, anticoagulants and statins), it is often deemed a liability.^[47]^ Ionisation to the carboxylate anion at physiological pH results in a diminished ability to diffuse passively across lipid cell membranes such as those in the intestine or blood brain barrier (BBB). Furthermore, metabolism of the carboxylic acid moiety *via* phase II glucuronidation reaction can complicate pharmacotherapy due to the production of idiosyncratic products.^[48]^

In works published by Lassalas investigating structure property relationships of carboxylic acid isosteres, it was determined that TZDs confer moderate acidity with an associated pKa value of around 6‐7 and is considered to be relatively lipophilic.[^49^, ^50^] This makes the TZD group prone to augmenting drug permeability *via* diffusion across biological membranes in comparison to its carboxylic counterpart. It is for these reasons that the TZD framework is considered a suitable surrogate.^[48]^ It should also be noted that many modulators of PPARγ feature the carboxylic acid moiety.^[51]^

### 3.2 TZDs as therapeutic agents

As discussed previously, TZD containing scaffolds are known to invoke a biological response across a wide variety of biological targets. The following section will deal with target specific interactions. Herein, both the mechanism of action brought about by the clinical agent as well as a discussion of the synthetic methodology used to generate the TZDs are included.

### 3.3 TZDs in the treatment of diabetes mellitus

The most common utilisation of TZD‐containing structures has been in the treatment of diabetes mellitus (DM), which is recognised as a metabolic disorder often characterised by hyperglycaemia and related ailments caused due to a deficiency in insulin secretion. There are two recognised forms of DM: type 1 (T1D) and type 2 (T2D). T1D is associated with either a total or partial lack of insulin brought about by a defect in the immune system resulting in a loss of tolerance and degradation (through inflammation) to the β‐cells. T2D is, however, associated with erratic degrees of insulin resistance, unregulated insulin secretion and an increase in hepatic glucose production hence sufferers are termed non‐insulin dependent.[^52^, ^53^] The concentration of glucose present in the blood is controlled *via* hormonal balance of both insulin and glucagon through a homeostatic mechanism. When glucose concentration is high there is an increase uptake of glucose by the skeletal muscle. In cases where the glucose concentration is low, glucagon promotes the synthesis and excretion of glucose.^[54]^ The International Diabetes Federation has predicted that the morbidity rate from diabetes will increase from 425 million in 2017 to 629 million by 2045.^[53]^

**Figure 5:**
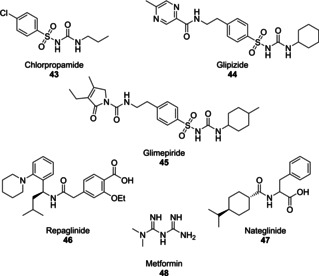
Commonly prescribed drugs used in the treatment of DM.

Conventional treatments for DM prior to the use of TZD based structures include drugs classes such as sulphonylureas (**43**–**45**), meglitinides (**46/47**) and biguanides (**48**) (Figure 5).^[55]^

#### 3.3.1 PPARγ inhibition

The first antihyperglycemic TZD that came to the attention of medicinal chemists was ciglitazone (**49**). Workers at the Takeda laboratories (Japan) successfully synthesised **49** in early 1975 while generating a series of 71 analogues of the hypolipidemic drug clofibrate with the aim of discovering a more potent derivative. They found that some of the products generated displayed hypoglycemic effects when tested on diabetic mice.^[56]^ Though it was initially approved by the US Food and Drug Administration (FDA), it was withdrawn from the market due to unacceptable liver toxicity.^[57]^ Then, troglitazone (**50**) was discovered and developed by Sankyo in 1988, and approved by the FDA for T2D treatments in 1997. However, within 6 weeks of its launch it was withdrawn from the UK market (much like **49**) as a result of potentially fatal hepatoxicity.^[57]^

**Figure 6:**
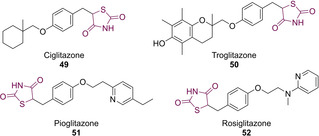
Marketed first generation glitazones featuring a TZD core.

In 1999, Takeda and SmithKline developed two TZD containing drugs, pioglitazone (**51**) and rosiglitazone (**52**) which were also approved by the FDA for the management of T2D (Figure 6).^[58]^ After capitalising the market for insulin sensitisers and becoming one of the top 25 selling brands in the United States, concerns were raised regarding rosiglitazone causing heart failure due to fluid retention and, in 2011, the European Medicines Agency recommended that it should be withdrawn from the market.[^59^, ^60^]

**Scheme 16:**
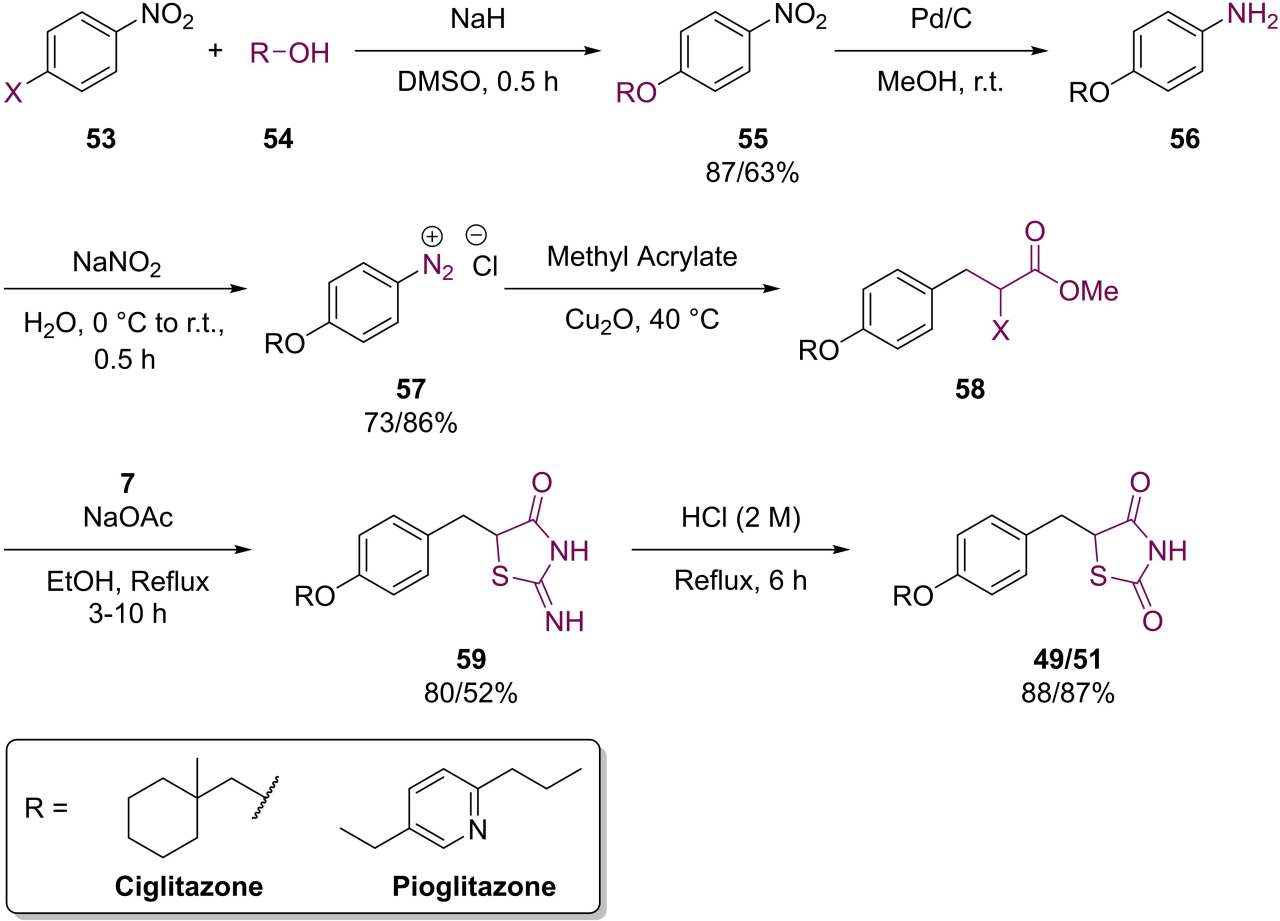
Initially utilised methodology to generate ciglitazone (**49**) and pioglitazone (**51**).

These ‘first generation’ ‘glitazones’ were initially prepared *via* condensation or nucleophilic substitution of a halogen substituted nitrobenzene (**53**) with an alcoholic precursor (**54**) to generate the intermediates, **55** (Scheme 16). These compounds were then subjected to hydrogenation conditions to provide the amines **56**. Diazotization of amine **56** was then conducted to yield the diazonium salts **57** which were condensed in the presence of methyl acrylate to afford the α‐halogenated esters **58**. The final steps of this synthesis introduced the TZD moiety *via* a cyclo‐condensation with thiourea (**7**) to afford the respective imines **59** before acidic work up and hydrolysis yielded **49** or **51** in relatively high yields (Scheme 16).^[56]^

**Scheme 17:**
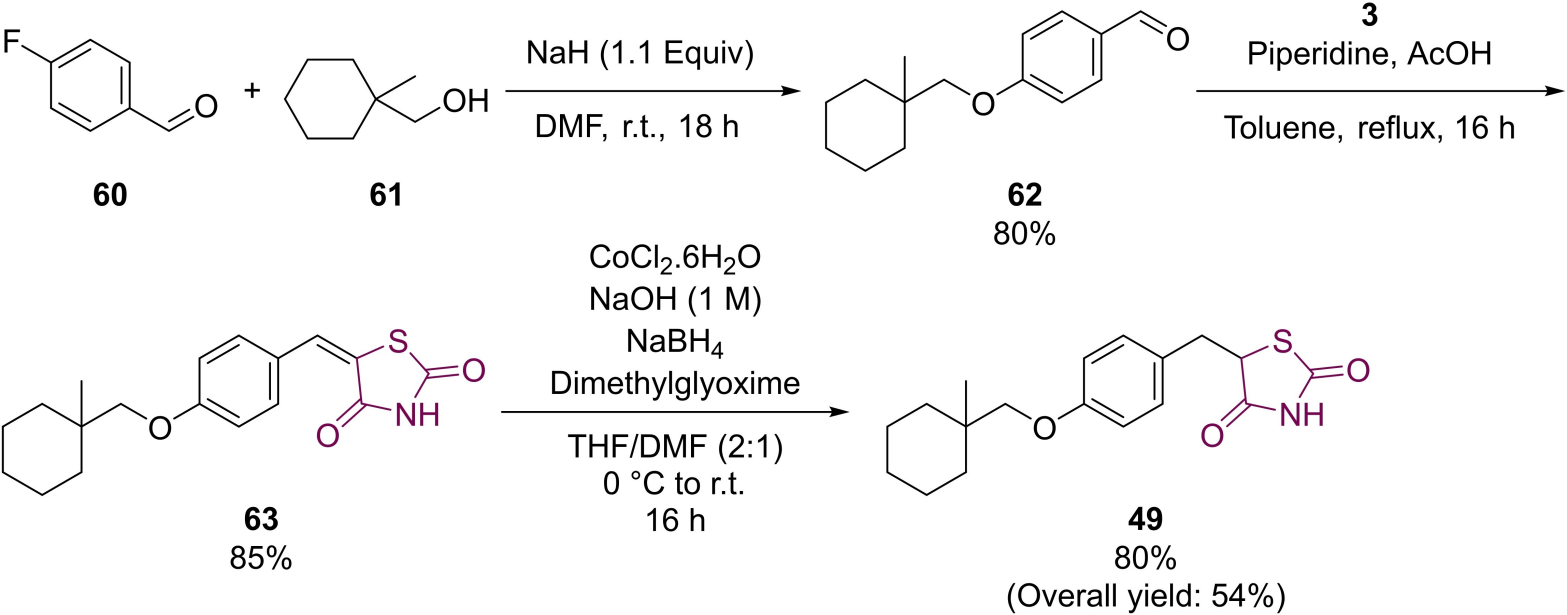
Process chemistry route to **49**.

Though suitable for small scale generation, the use of pyrophoric NaH, expensive metal catalysts, toxic acids as well as potentially explosive intermediates being generated may result in it being unsuitable for significant upscaling. Therefore a process chemistry methodology was developed which made use of commercially available TZD.^[61]^ The improved methodology synthesised **49** in a 54 % overall yield over a series of three steps (Scheme 17).

In the above synthesis, substitution of the commercially available *p*‐fluorobenzaldehyde (**60**) with **61** in the presence of NaH, in DMF, afforded **62** which was, in turn, coupled with TZD (**3**) *via* a KC to generate the penultimate intermediate (**63**). The final step in the synthesis required a reduction of the benzylidene olefin which was achieved using NaBH_4_ in the presence of NaOH.^[61]^

PPARs are ligand inducible transcription factors that are important biologically for cell differentiation, lipid and glucose homeostasis, insulin sensitivity, inflammatory responses and various other metabolic processes.^[62]^ Such receptors are known to exist in a series of three distinct isoforms namely PPARα, PPARβ/δ and PPARγ.^[63]^ Agonist binding within the ligand binding region (LBR) cause a translocation to the cell nucleus and heterodimerisation with retinoid X receptor (RXR), another nuclear receptor. Following dimerisation they bind with a specific region of DNA known as peroxisome proliferator hormone response elements (PPREs).^[64]^ PPARα and PPARγ are greatly expressed in the heart, liver and skeletal muscle as well as in adipose tissue respectively.^[63]^

Binding of TZDs promotes fatty acid uptake and subsequent storage in adipose tissue. As a result, fat levels are reduced in the liver, pancreas and muscles, leading to protection from toxic products formed by metabolism of free fatty acids. Furthermore, TZDs interfere with cellular signalling pathways between insulin sensitive tissues and organs such as the liver, muscle and adipose tissue. They have also been seen to increase the production of adipokines which are insulin sensitisers.^[65]^

**Figure 7:**
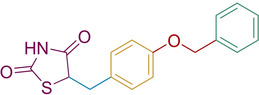
Simplified pharmacophore associated with PPARγ inhibitors.

Through extensive structure activity relationship (SAR) studies conventional TZDs can be split into five common moieties; an acid TZD head group (purple), a lipophilic tail (green), linked to a central phenyl ring (orange) by two aliphatic chains (blue/red) (Figure 7).

Agonist activity towards PPARγ is usually brought about through the formation of hydrogen bonds with the P_1_ hydrophilic binding pocket present in arm I of the active site.^[66]^ Specifically, hydrogen bonding interactions are formed with His323, His449, and Tyr473. The lipophilic tail (green) would react with binding sites present in both arm II and arm III through a multitude of hydrophobic interactions, including VDW and π‐π stacking interactions. The aliphatic linking chains (blue/red) act as spacers in order to orientate the acidic TZD head (purple) and lipophilic tail (green) into their binding pockets. Finally, the phenyl ring present in the centre (orange) interacts with further hydrophobic amino acid residues in the cleft in a similar way to that of the tail group.^[67]^

**Figure 8:**
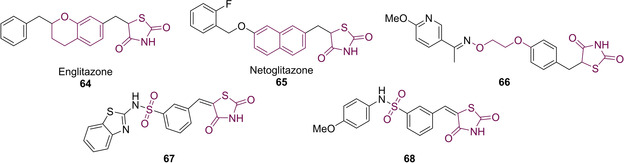
Modifications to the central phenyl ring and linker region of PPARγ modulators.

Modifications of the blue‐coloured alkyl linker (Figure 7) were tolerated, providing that the alkyl chain did not exceed three carbon atoms.^[66]^ It is likely that this linker orientates the molecule and provides the required space between the lipophilic tail and polar acidic head group. The central phenyl ring is seen to be highly significant in the binding interactions to bring about PPARγ agonism though substitution with a benzdihydropyran and naphthyl groups (englitazone (**64**) and netoglitazone (**65**) respectively) are also well tolerated. The second linker (red) has also been subjected to structural modifications. The severe adverse side effects brought about by **49** have been attributed to the fact that it features a very small aliphatic linker of just one carbon atom which fails to present **49** in the desired conformation for efficient binding and often results in off target interactions.^[66]^ A series of analogues are shown below with modifications to this alkyl linker. Compound **66** features the maximum number of six atoms between the central phenyl ring and the lipophilic tail, it is thought that the oxime functional group plays a vital role through the provision of hydrophobic interactions.^[66]^ A more exotic linker was introduced using a sulfonyl group (**67**/**68**) which most likely behaves as a weak HBA resulting in the formation of a short *syn* orientated hydrogen bonds planar to the axis of the S=O group with residues present at the entrance of the ligand binding domain (LBD) (Figure 8).^[68]^

**Figure 9:**
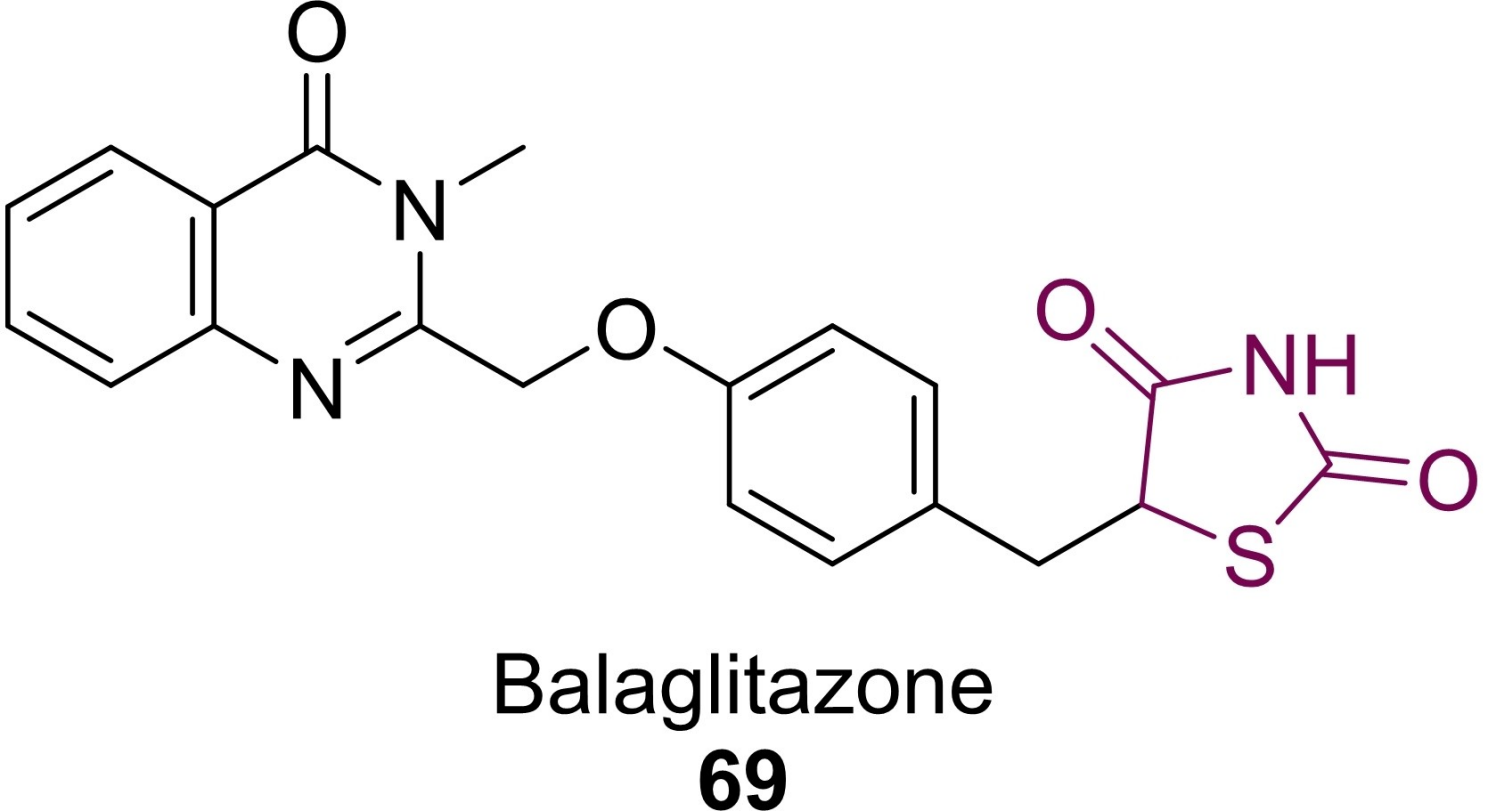
Balaglitazone: a currently used PPARγ modulator.^[66]^

The final structural region within the general pharmacophore model which can receive structural modification is the green lipophilic tail (Figure 7). During drug‐receptor interactions, the affinity of the drug is determined predominantly by the extent of hydrophobic interaction.^[69]^ It has been evaluated that within the LBD of PPARγ there exists two large hydrophobic pockets, namely P_3_ and P_4_ respectively.^[66]^ It is for this reason that large hydrophobic units are required in the tail end of suitable drug candidates. Rosiglitazone (52) possesses a large sized pyridine ring as the tail but is unable to reach both hydrophobic pockets, only reaching P_3_. On the other hand, balaglitazone (69) features a bulky benzopyrimidinone group as the hydrophobic tail which is shown to be a partial agonist of PPARγ. It is believed that partial agonist activity is brought about by hydrophobic interactions with both pockets P_3_ and P_4_ (Figure 9).^[66]^

**Figure 10:**
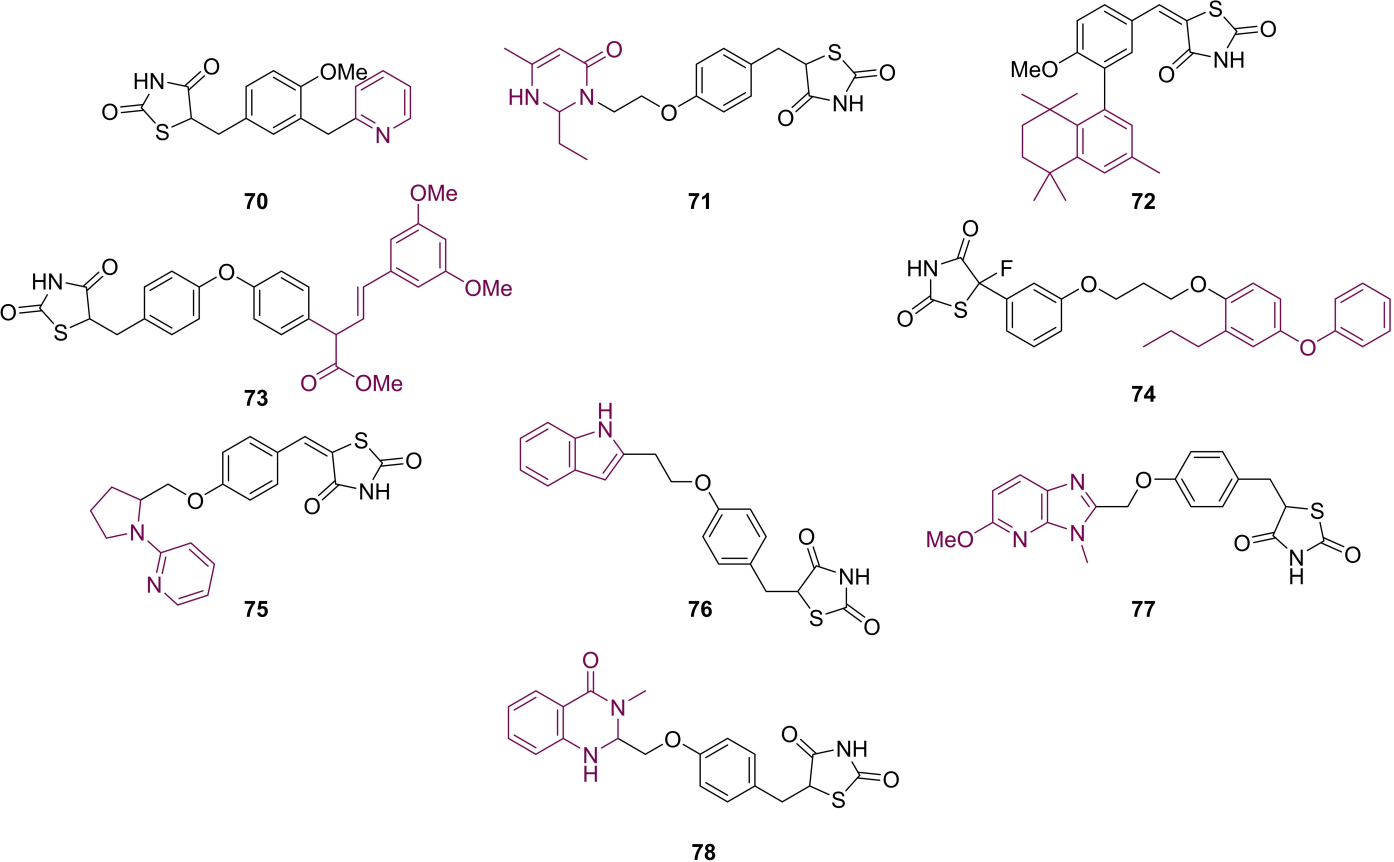
Modification to the lipophilic tail of PPARγ inhibitors.

Exchange of the lipophilic tail with pyridyl (70) and pyrimidyl (71) moieties showed an increase in agonist activity at the micromolar level with pyridyl conferring slightly higher potency. In the case of 71, a decrease in plasma glucose and triglyceride levels of 73 % and 85 % respectively was observed.^[70]^ Such moieties exhibit better agonist activity than both 51 and 52 (first generation glitazones) in terms of both oral absorption and less severe side‐effects.^[70]^ PPARγ inhibition was also witnessed when the lipophilic tail was substituted with tetrahydronaphthalenes (72), styryl (73)^[71]^ and diphenyloxy (74) groups and by a range of nitrogen‐containing heterocycles (75–78) (Figure 10).[^72^, ^73^, ^74^]

#### 3.3.2 PTP1B inhibition

A second well recognised therapeutic strategy for the treatment of DM is through the inhibition of protein tyrosine phosphatase 1B (PTP1B). The phosphorylation‐dephosphorylation of biological molecules is recognised as a vital mechanism in the control of cellular function, growth, communication and differentiation.^[75]^ Protein tyrosine phosphorylation also plays an active role in transmitting extracellular responses which can lead to T‐cell activation and antigen‐receptor signalling pathways. The process is led by two opposing enzyme; kinases (PTK) which conduct phosphate transfer, and, protein tyrosine phosphatases (PTP) which act to catalytically hydrolyse the previously installed phosphate group.^[76]^ PTP are distinguished in terms of structure by the presence of only one catalytic domain consisting of around 240 residues with arginine and cysteine acting as essential amino acids in the catalytic process.^[77]^ *In vivo*, PTP maintain the level of tyrosine phosphorylation which has shown an association with T2D.^[77]^

PTP1B is recognised to be a negative regulator of insulin‐receptor and leptin‐receptor signalling pathway. The binding of leptin to its receptor results in phosphorylation of Janus Kinase 2 (JAK2) and further activates the associated JAK signal transducer and activation transcription (STAT). This results in translocation of STAT3 to the cell nucleus and subsequently induces a gene mediated response to reduce the production of acetyl coenzyme‐A carboxylase (ACC). ACC is a biotin‐dependent enzyme which catalyses the conversion of acetyl coenzyme A (Ac‐CoA) to malonyl CoA, this in turn halts or reduces the rate of fatty acid synthesis; while increasing the rate of fatty acid oxidation *via* metabolism.^[78]^

**Scheme 18:**
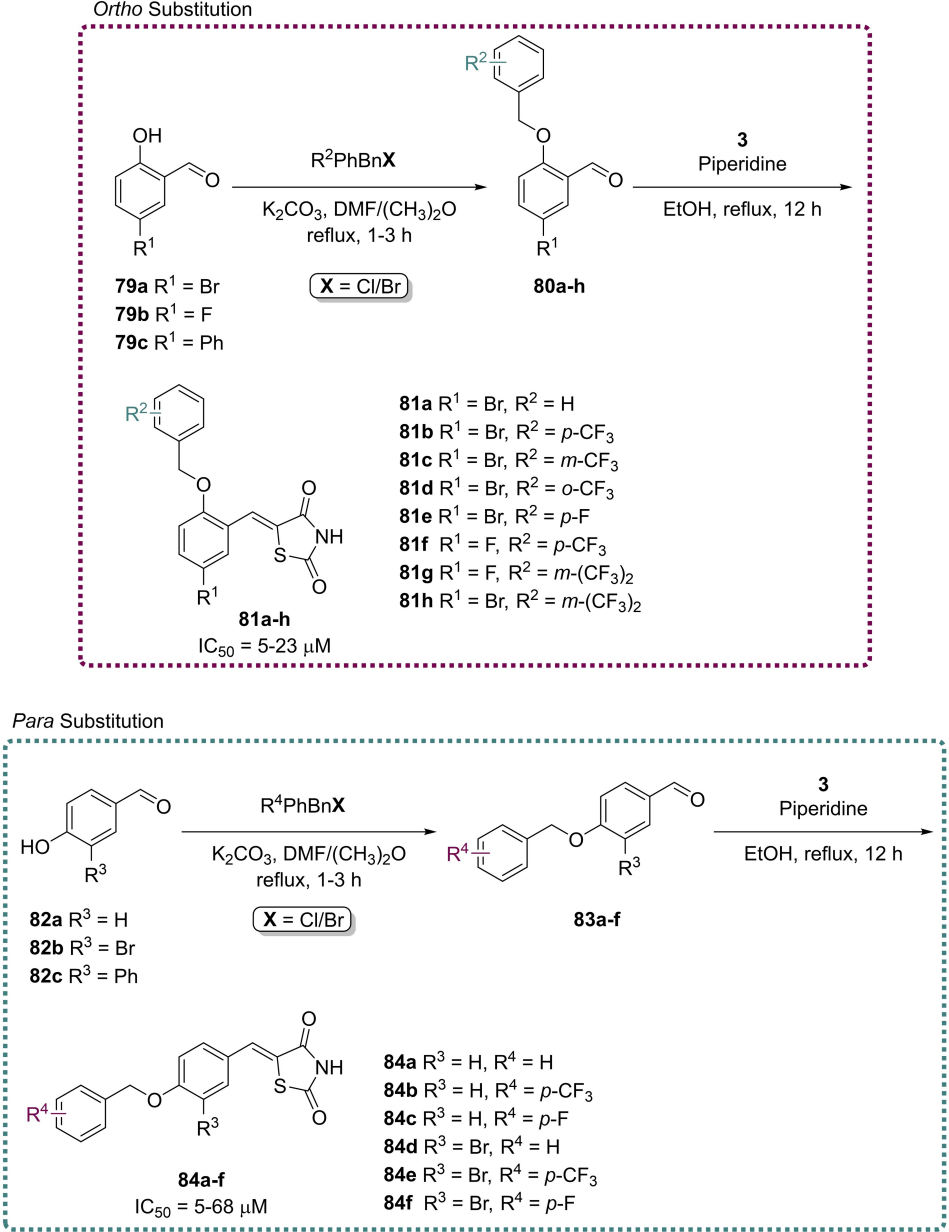
Benzyloxy‐substitutions at ortho/para sites of 5‐benzylidene PTP1B inhibitors.

In an effort to develop a series of biologically active compounds, Bhattarai and co‐workers initially looked to the previously described PPARγ modulators (glitazones) to assess their inhibition towards PTP1B. They found that clinically used glitazones **50**–**52** displayed medium to low potency in terms of PTP1B inhibition with IC_50_ values between 55–400 μM. Glitazones which feature substitution at the C_5_ position with a benzylidene linker are termed 5‐benzylidene‐TZD derivatives. Using these glitazones as a scaffold for their subsequent research, the group investigated altering the location of the benzyloxy group stemming from the central phenyl ring to develop a series of analogues where substitution occurs at the *ortho* and *para* positions (Scheme 18).^[79]^

**Scheme 19:**
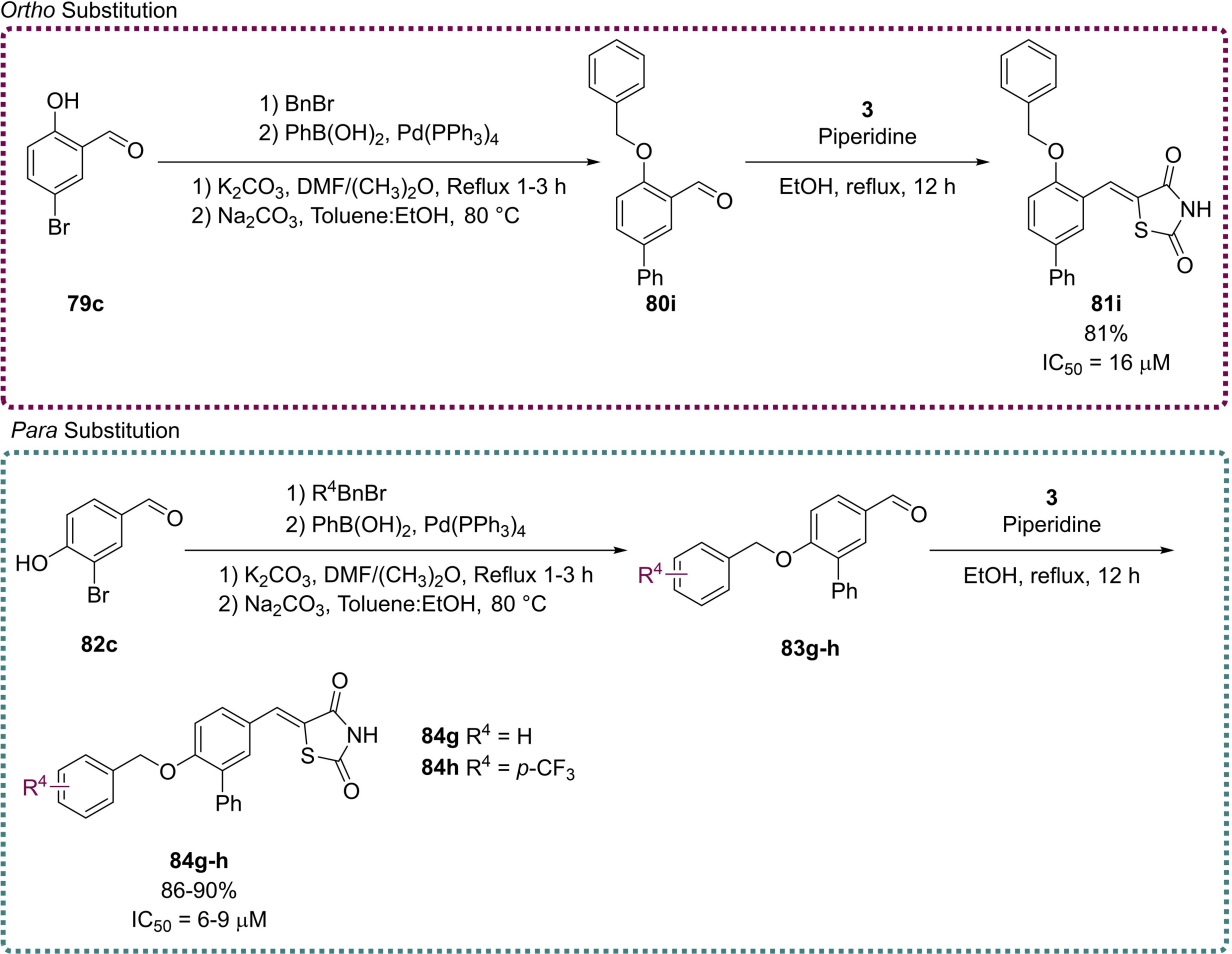
Suzuki coupling reactions to generate ortho/para substituted 5‐benzylidene PTP1B inhibitors.

The derivatives illustrated in Scheme 18 were synthesised *via* the heavily reported method of conducting a KC in the presence of a piperidine catalyst. In cases where this methodology did not yield the desired product (**81 i**, **84 f** and **84 g**), the Nobel prize winning Pd cross‐coupling Suzuki reaction was employed to generate **81 i** and **84 g**–**h** in yields of 81 %, 90 % and 86 % respectively (Scheme 19).^[79]^

Following synthesis, the analogues generated were tested for *in vivo* inhibition of PTP1B in an obese and diabetic mouse model (C57BL/6J Jms Slc, male). Bhattarai found that all of the generated compounds were significantly more potent than the marketed glitazones. Substitution at the *ortho* position brought about a higher level of inhibition with IC_50_ values ranging from 5–23 μM. The most potent analogues synthesised were **81 h** and **84 e** (Schemes 18 & 19), both possessing an IC_50_ value of 5.0 μM.^[79]^

Analogue **81 h** was further evaluated in order to obtain an insight into its inhibitory mechanism of action. In a series of docking simulations, it was illustrated that a pair of hydrogen bonding interactions are made from Gln266 and the backbone amino group of Ser216 to the carbonyl oxygen substituents on the TZD frame.^[79]^ The acidic proton occupying the amidic position was assumed to be deprotonated *in vivo* due to it possessing a p*K*_a_ value of ∼6.74. In its deprotonated state, **81 h** is seen during autodock simulations to be close to Cys215 and Arg221 with a distance between them of within 4–5 Å. Further interaction (and hence stabilisation) within the catalytic site can be seen through hydrophobic interactions between the inhibitor's aromatic rings and surrounding hydrophobic residues, including Tyr46, Val49, Phe182, Ala217 and Ile218. In the same *in vivo study*, **81 h** was administered orally with food at 143 mg/day/Kg (of weight) for a period of 4 weeks which showed a reduction in both weight and glucose tolerance. Significantly lower levels of total cholesterol and triglycerides were present in the serum of mice consuming **81 h** though dark brown spots were observed in the liver (suggesting liver damage) indicating that **81 h** required further optimisation through derivatisation.^[79]^

The exhibited side‐effects as described when utilising the general structure for PPARγ inhibitors has since been attributed to off target interactions from PTP1B. Specifically, the acidic nature of the proton attatched to the *N*_3_ position on the TZD frame is important. This rationale comes from its resemblance to the carboxylate anion of the natural fatty acid ligands which are known modulators for PPARγ. Due to these findings there has been a push for the development of novel *N*‐substituted TZD derivatives.^[80]^ A reduction in target promiscuity should provide a reduction in side‐effects, specifically relating to hepatotoxicity. It should be noted that Bhat *et al*. has since utilised these findings to successfully develop a series of *N*‐substituted glitazones with little to no observed toxicity.^[81]^

**Figure 11:**
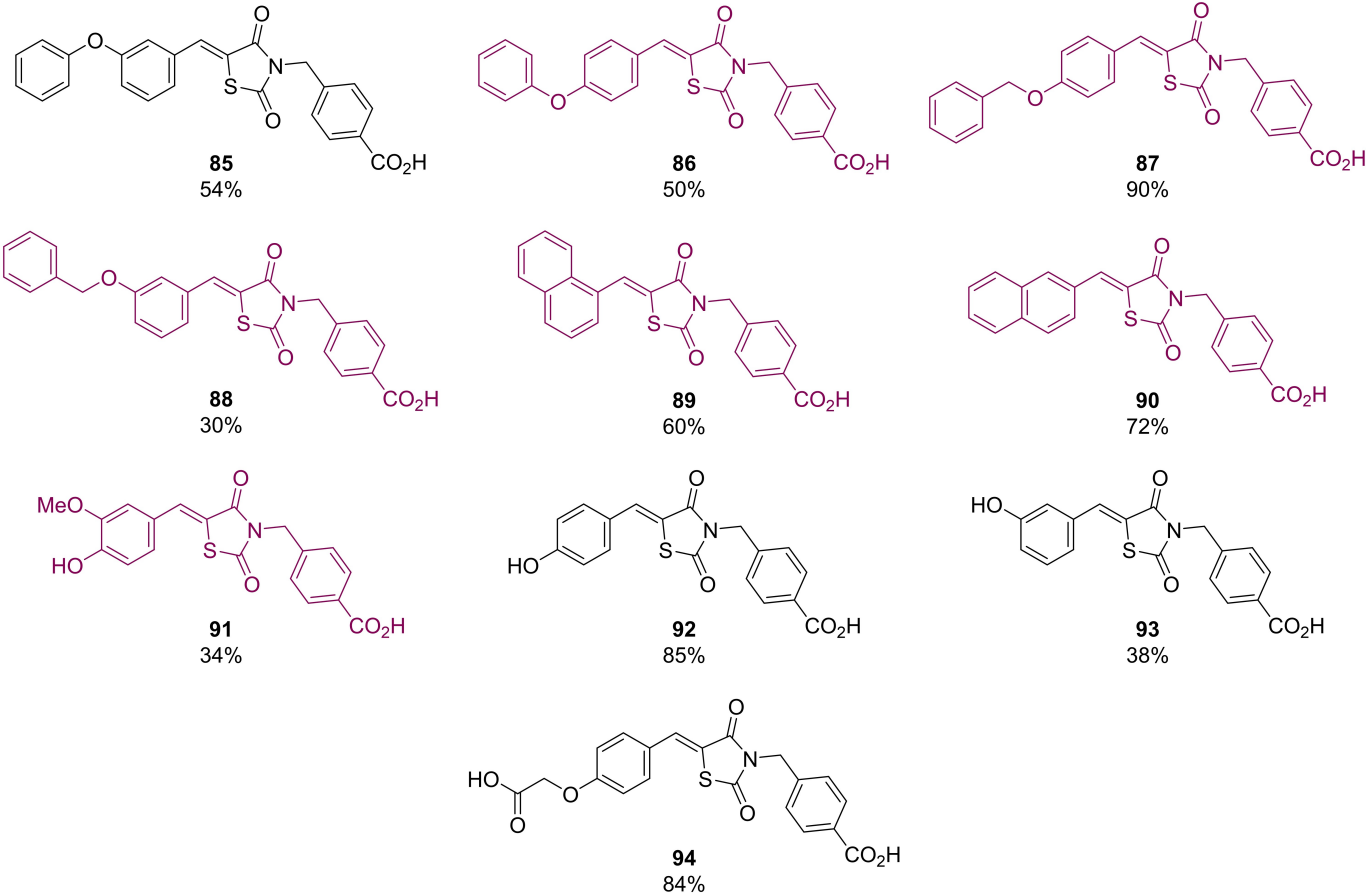
5‐benzylidene *N*‐para‐benzoic acid analogues for PTP1B inhibition.

In 2007, Maccari and colleagues set about developing a series of *N*‐substituted 5‐arylidene derivatives as modulators to PTP1B.^[82]^ They began with the installation of a *para* methylbenzoic acid group onto the *N*‐3 position of TZD. This was primarily due to the fact that benzoic acid acts as a phospho‐tyrosine isostere.^[83]^ In their published work they successfully synthesised a series of 10 analogues (Figure 11). Installation of groups at the methylene position proceeded through the already discussed KC with the corresponding aromatic aldehyde and TZD, in the presence of a piperidine catalyst, in refluxing EtOH. Substitution at the amidic position was achieved by refluxing the 5‐substituted TZD in 4‐bromomethyl benzoic acid, in the presence of K_2_CO_3_, prior to acidic workup and recrystallisation from hot MeOH. The synthesised analogues were evaluated for activity *in vitro* against recombinant human PTP1B as well as the two present active isoforms. Compounds **86**–**91** were shown to exhibit PTP1B inhibition with IC_50_ values in the low micromolar range (1.1–6.5 μM). Within this subset, compound **86** and **87** displayed the most effective inhibition to both isoforms of PTP1B.^[82]^

**Figure 12:**
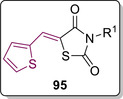
General structure for thiophene substituted TZD derivatives.

In 2017, Mahapatra helped to tackle a common problem associated with 5‐arylidene TZD based compounds.^[84]^ Classically, active site directed PTP1B modulators have possessed a high charge density which brings about a host of issues in terms of pharmacokinetics and poor membrane permeability. This, in turn, reduces oral bioavailability and drug‐likeliness.^[85]^ To overcome these problems, Mahapatra looked at exchanging the arylidene substitution at the methylene position, and turned to inspiration from work published by Anderson, Moretto and Ye.[^86^, ^87^, ^88^] Their work has shown positive results towards PTP1B inhibition *via* the inclusion of thiophene based compounds. Mahapatra therefore envisaged a series of small molecule inhibitors containing a TZD core, *N*‐substitution with a lipophilic alkyl or haloalkyl group and a thiophene derivative installed at the methylene position joined to TZD *via* a vinyl linker (**95**). The general structure for this series of compounds can be seen below in Figure 12.^[84]^

**Scheme 20:**
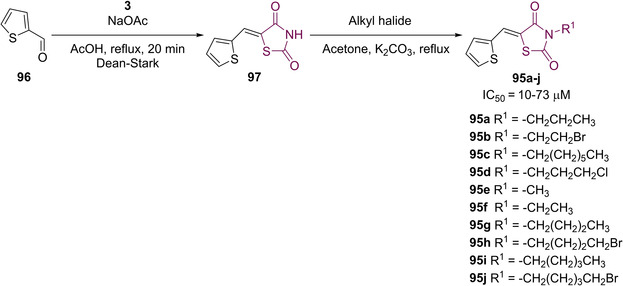
Synthesis of analogues **95 a**–**j**.

A series of 10 *N*‐alkyl/alkyl halide analogues were synthesised (**95 a**–**j**) in good to high yield (59–86 %) via KC of TZD (**3**) with thiophene‐2‐carboxaldehyde (**96**), to afford **97**, prior to *N*‐alkylation with the appropriate mono/disubstituted alkyl halide utilising anhydrous K_2_CO_3_ as a base (Scheme 20).^[84]^

*In vitro* studies utilising the BML‐AK822 assay kit containing human, recominant PTP1B (residue 1–322) expressed in *E*. coli revealed binding affinities ranging from 10–73 μM with smaller substituents possessing greater potency. The highest potency was witnessed with **95 e** (IC_50_=10 μM) while the lowest potency was exhibited by **95 c** (IC_50_=73 μM). As seen with previous examples, the carbonyl group present on the TZD core partakes in a hydrogen‐bonding interaction with Arg221 as well as previously unwitnessed interactions with Lys120 (a residue present in the catalytic cleft of PTP1B). Further interactions were observed through π‐π stacking of the thiophene ring and Tyr46. Alongside their SAR studies, the group conducted full computational predictions on the pharmacokinetics parameters for each analogue generated. All of the compounds present no violations with reference to Lipinski's guidelines.^[84]^

#### 3.3.3 ALR2 inhibition

DM has also been associated as a leading cause of new cases of partial vision loss or total blindness, as well as health concerns relating to heart disease, neuropathy and nephropathy.^[89]^ Diabetic retinopathy is characterised by capillary cell loss, capillary membrane thickening in the basement reagent and an increase in leukocyte adhesion to endothelial cells.^[89]^ Such medical conditions are brought about through complications in glucose metabolism involving the Aldose reductase 2 enzyme (ALR2) in the polyol pathway.^[90]^

**Scheme 21:**
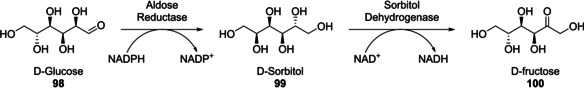
The role of ALR2 in the polyol pathway to generate fructose from glucose.

ALR2 is an enzyme belonging to the aldo‐ketoreductase superfamily which during the polyol pathway catalyses the NADPH‐induced reduction of glucose (**98**) to sorbitol (**99**). Following this reduction, sorbitol is subjected to an oxidation reaction by sorbitol dehydrogenase generating the pentose sugar fructose (**100**) (Scheme 21). In healthy humans, only a small amount of glucose is metabolised *via* this pathway as the majority undergoes a phosphorylation reaction *via* hexokinase to generate the hexose sugar glucose‐6‐phosphate which is, in turn, used as a substrate for glycolysis, a key process in cellular respiration.^[91]^ In cases of chronic hyperglycaemia (such as those suffering with DM), stimulation of the polyol pathway is significantly increased. As ALR2 is highly prevalent in the cornea, retina and lens as well as within the kidneys and neurone myelin sheaths, the aforementioned medical complications are usually witnessed.^[92]^

**Figure 13:**
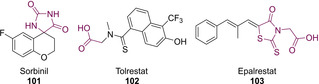
Clinically used ALR2 inhibitors.

The two main classes of ALR2 inhibitors are cyclic imides such as sorbinil (**101**) and epalrestat (**103**) (usually containing hydantoins), and carboxylic acids such as tolrestat (**102**) (Figure 13). Though carboxylic acids have been shown to exhibit high *in vitro* potency they are generally less active *in vivo* compared to imides. This could be attributed to the vast extent of metabolism associated with carboxylic acid‐containing compounds *via* phase II conjugation reactions.^[93]^ Members of this functional group series exhibit acidity and less favourable pharmacokinetic properties.^[94]^ There is a need to further develop series of cyclic imide based compounds with a hope to remove the toxicity associated with the hydantoin moiety. One such strategy has involved the use of the TZD framework because it is a known and recognised bioisostere for hydantoins.^[95]^

**Figure 14:**
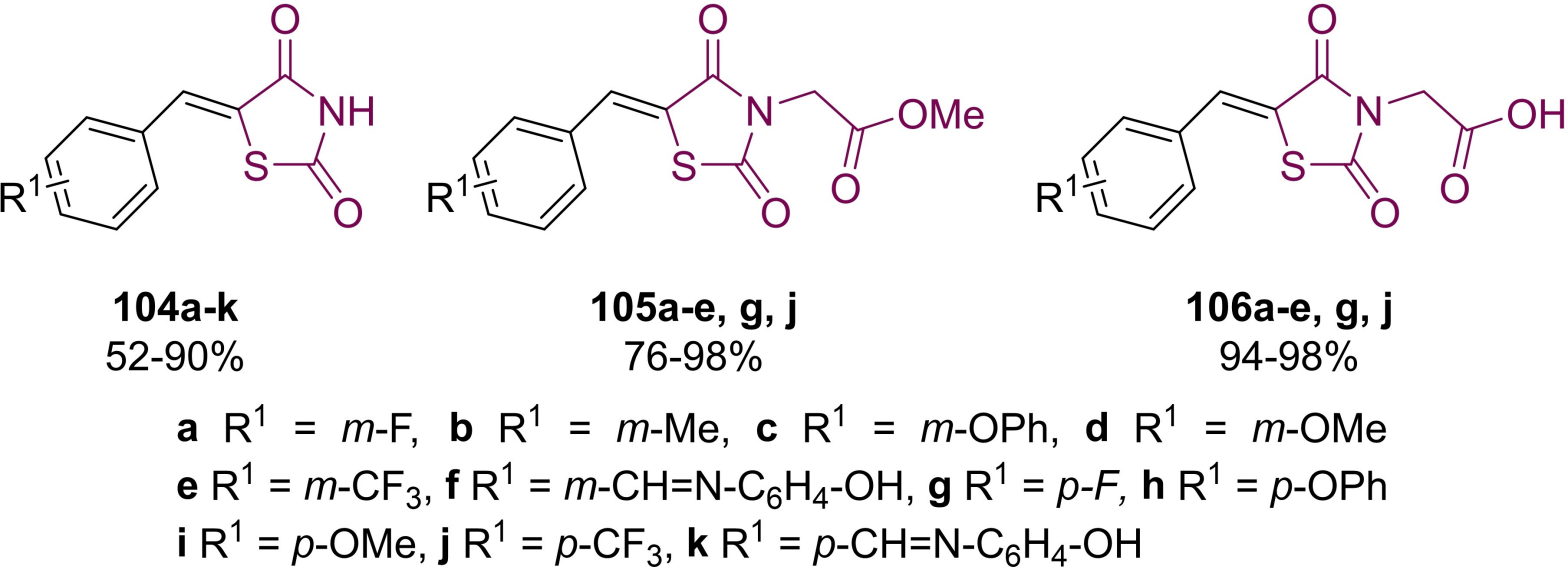
5‐arylidene analogues generated by Maccari.^[96]^

With these considerations in mind, and in an effort to develop a series of novel inhibitors for ALR2 containing a TZD core, Maccari and co‐workers synthesised a distinct set of three TZD‐containing 5‐arylidene TZDs. Their work followed on from previous published work which outlined the necessary pharmacophore for successful ALR2 inhibition.^[96]^ This pharmacophore requires the presence of an acidic proton, the ability to act as a HBA and to contain a substituted aromatic ring.[^97^, ^98^] The first series of compounds generated (**104 a**–**k**) possessed the acidic amidic hydrogen on the TZD core, the second series generated consisted of replacing the acidic hydrogen with an acetate ester group (**105 a**–**e**, **g**, **j**), while the third series saw generation of the corresponding acetic acid (**106 a**–**e**, **g**, **j**) (Figure 14).^[96]^

Synthesis of analogues possessing the general structure **104** was achieved *via* KC of TZD (**3**) with the corresponding *meta*/*para*‐substituted benzaldehyde utilising piperidine as a catalytic base. Deprotonation of the acidic proton was achieved with NaH in DMF before the generation of the ester **105** with methyl bromoacetate. Subsequent hydrolysis afforded the appropriate carboxylic acid (**106**) *via* an acetic acid‐catalysed process.^[96]^

Analogues featuring the general structure **104** showed the greatest variation in yield. Compounds **104 f**/**k** were produced in the highest yields of 88 % and 90 % respectively. Lower yields were witnessed (52 % and 57 %) in cases featuring a strong EWG fluorine or trifluoromethyl at the *meta* position (**104 a**/**e**). Likewise, a lower yield of 52 % was achieved when a methoxy unit was introduced at the *meta* position (**104 d**). In the case of molecules featuring the general structure **105** significantly higher yields can be witnessed. The highest yield was generated in the case of *meta* methoxy‐derivative **105 d** (98 %) closely followed by *meta* substituted fluoride **105 a** (96 %). Very high yields were achieved in all cases (**106 a**–**e**, **g**, **j**) when the ester was hydrolysed to the corresponding carboxylic acid.

The potency of compounds featuring an acidic group has been deemed highly important for future work in the development of ALR2 inhibitors. This acidic proton is considered to be ionised at physiological pH and will form ionic interactions with the active site of the enzyme.^[96]^

In 2005, Maccari *et al*. conducted further optimisation of their previous work by running a series of molecular modelling studies in order to generate a comprehensive understanding of SARs.^[99]^. They found that the presence of a second aromatic ring on the 5‐benzylidene group increased potency as compared to molecules which only possess one aromatic ring. Furthermore, substitution at the *meta* position showed an increase in activity which was independent of the nature of the substitution. In terms of *N*‐functionalisation, they found that the presence of an acetate chain caused an increase in affinity which they attributed to the fact that a polar interaction is formed with Tyr48, His110, Trp111 as well as the nicotinamide ring of NAD^+^.^[99]^

**Scheme 22:**
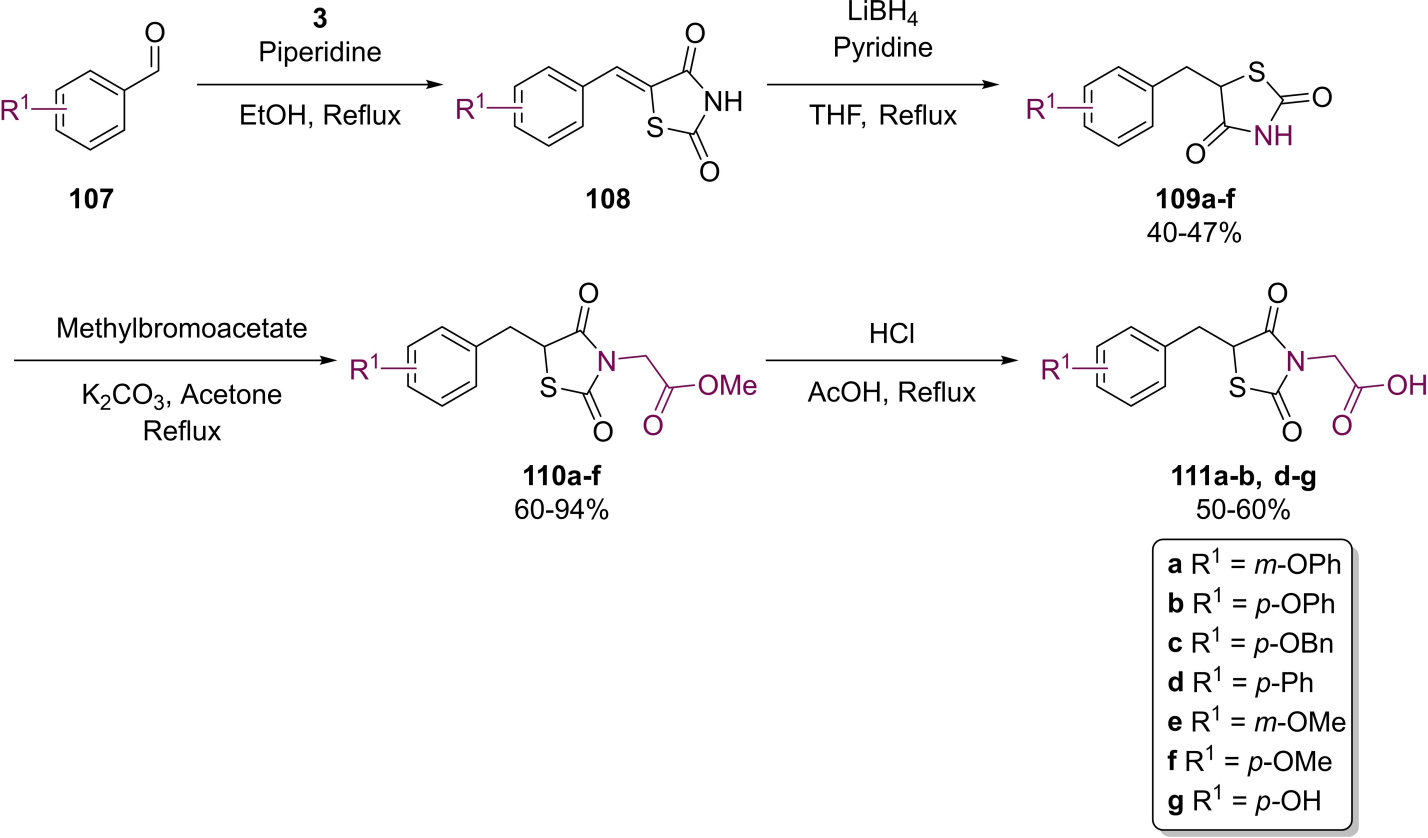
Synthesis of benzyl substituted ALR2 inhibitors by Maccari.^[100]^

With an interest in optimising potency even further, Maccari and colleagues investigated the effects of reducing the 5‐arylidene olefin present in their substrates to the corresponding benzyl derivatives.^[100]^ The group managed to successfully synthesise a further eighteen analogues to explore the effect of no substituent on the nitrogen atom and substituting it with both the acetate ester (as seen in **105**) and a carboxylic acid (**106**) (see Scheme 22). Reduction of the benzylidene group was achieved through the addition of LiBH_4_ in pyridine as per the procedure reported by Giles which showed selective hydrogenation of the olefin.^[101]^

Among the series of *N*‐unsubstituted derivatives, **109 a**–**d** displayed IC_50_ values ranging from 31–79 μM in an *in vitro* bovine lenses’ assay towards ALR2. Despite this moderate to poor potency derivative **109 b**–**d** showed a significant increase in potency as compared to the previously generated benzylidene analogues which, at a concentration of 50 μM, only generated 41 %, 10 %, and 20 % inhibition respectively. This increase in activity however was not apparent when the phenoxy substituent was installed in the *meta* position nor was it observed when the phenoxy was replaced with a methoxy unit. Among the analogues generated, which featured methyl esters, only **110 e** displayed ALR2 inhibition (IC_50_=21 μM). While this is a 2.5‐fold increase compared to the *N*‐unsubstituted derivative it is significantly less effective than the corresponding acid **111 e**. Finally, the carboxylic acid bearing products displayed a 15‐ to 80‐fold increase in potency as compared to their analogous benzylidene derivatives.^[100]^

**Figure 15:**
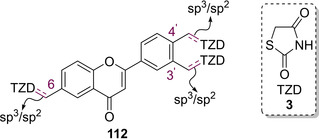
General structure of flavone substituent coupled onto TZD.^[102]^

Inspired by the works of Maccari, Bozdağ‐Dűndar *et al*. also looked to develop a series of ALR2 inhibitors featuring a TZD core in 2008. They substituted the benzylidene moiety commonly used in clinical agents to treat DM for flavonoid based systems.^[102]^ Flavonoids are recognised as a ubiquitous motif present in a wide range of edible, plants, fruits and plant‐derived beverages (including juices and teas). They have also been deemed as health‐promoting and disease preventing motifs which have seen use as antibacterial and antiviral agents.[^103^, ^104^] The group synthesised a series of ten flavone‐substituted TZDs and separated them into three distinct classes depending upon where the flavone unit was coupled to the methylene carbon. In the series of analogues generated substitution occurred at either the 3’, 4’ or 6 position (see Figure 15).^[102]^

**Figure 16:**
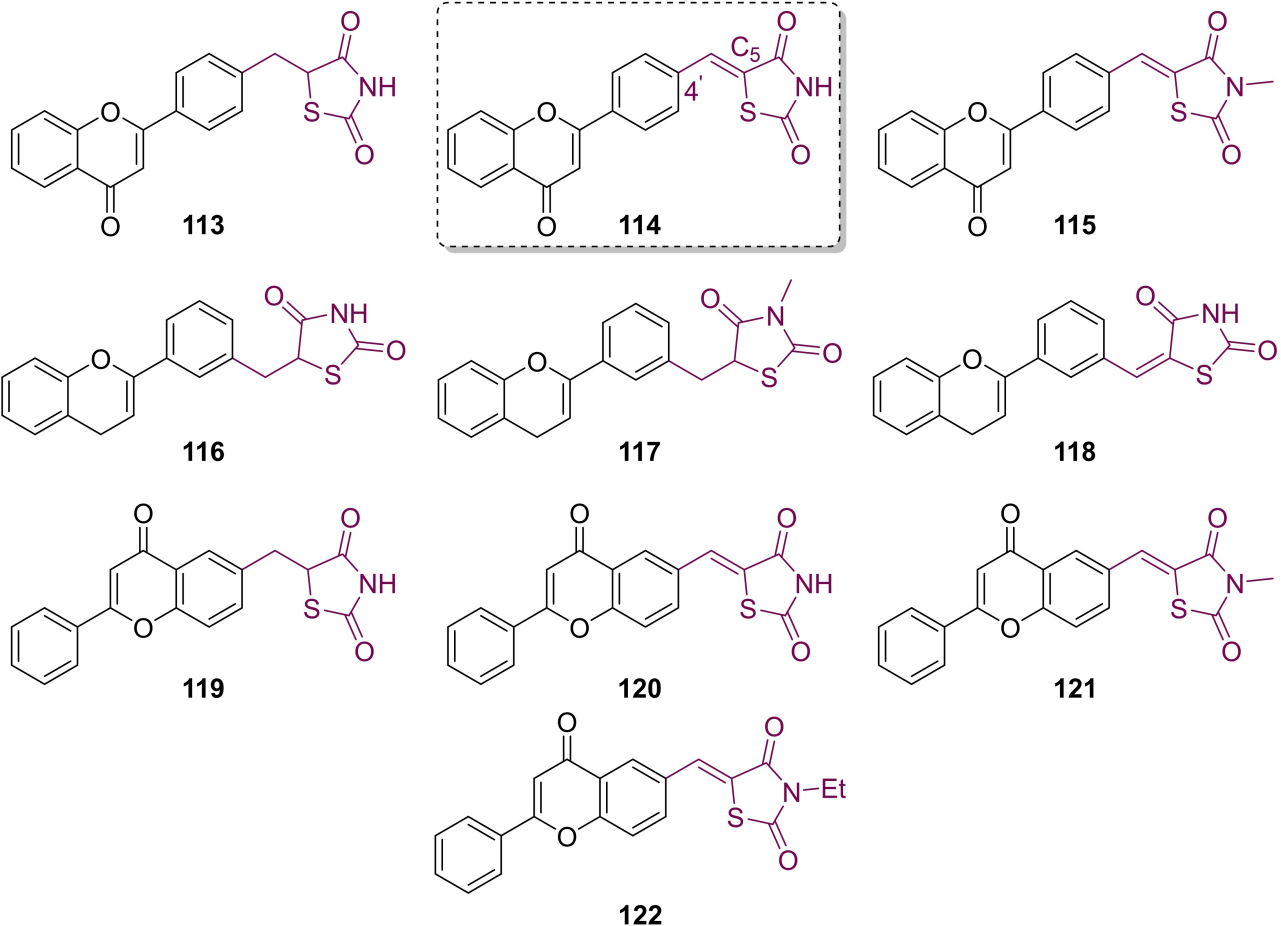
Flavone‐substituted TZD derivatives.

Along with altering the position of substitution onto the flavone ring, the group also explored developing linkers featuring sp^3^ and sp^2^ hybridised carbons and substances featuring both *N*‐substitution and no substituent on the nitrogen atom. Analogues without the presence of an olefin (**113**, **116**, **117**, **119**) were generated through the coupling of the appropriate bromomethylflavone with dilithio‐TZD, and the rest of the structures were generated *via* KC with the corresponding 3’/4’/6‐carboxaldehydes in the presence of AcOH and NaOAc. Substitution at the *N*_5_ position was carried out with the aid of an alkyl iodide under basic conditions (Figure 16).^[102]^

After conducting a series on *in vitro* experiments to assess potency, it was found that substitution at the 4’ position yielded the highest inhibitory activity. The most active analogue generated was **114** which possessed a potency of 0.43 μM. Substitution at the *N*_3_ position with a Me group showed some activity but still less than unsubstituted derivatives. Further, the presence of a double bond at the C_5_ position did not significantly impact potency.^[102]^

**Scheme 23:**
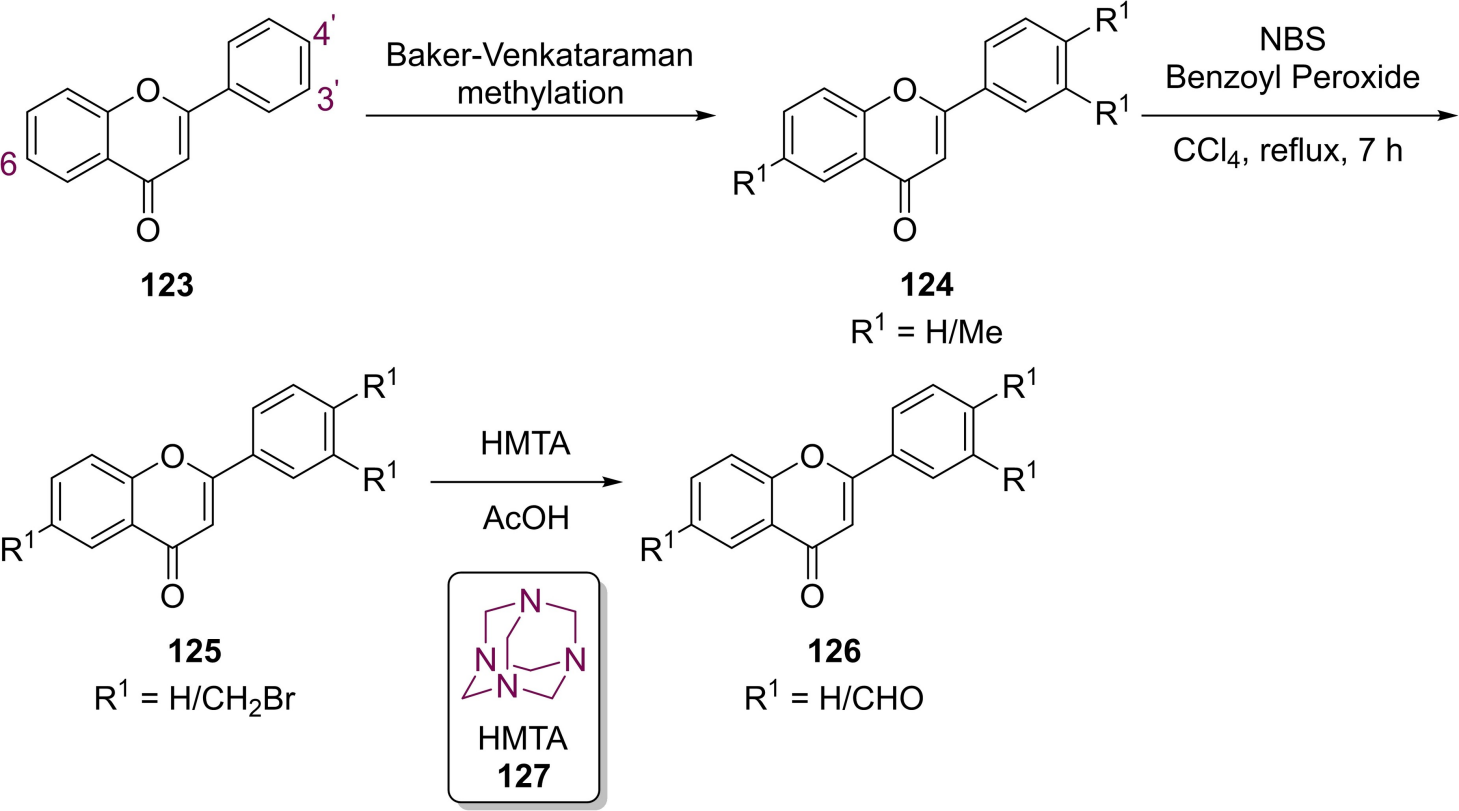
Synthesis of precursors for the generation of TZD analogues featuring flavone substitution.

In the same year, Bozdağ‐Dűndar published an additional paper on the generation of flavone‐substituted TZDs. All analogues generated in this study featured the olefin linker between the TZD core and the flavone substitution, and substitution at the *N*_3_ position appeared in the form of acetyl esters or acetic acids. In order to generate the 3’/4’/6‐carboxaldehyde precursors required for later KC, a methyl substituted flavone initially had to be prepared.^[105]^ This was completed using the Baker‐Venkataraman method (**124**).^[106]^ Subsequent bromination utilising NBS and a catalytic quantity of benzoyl peroxide afforded **125**. The final aldehyde was generated *via* the addition of HMTA (**127**) in an acidic environment by means of a Sommelet reaction, generating **126** (Scheme 23).^[105]^

**Figure 17:**
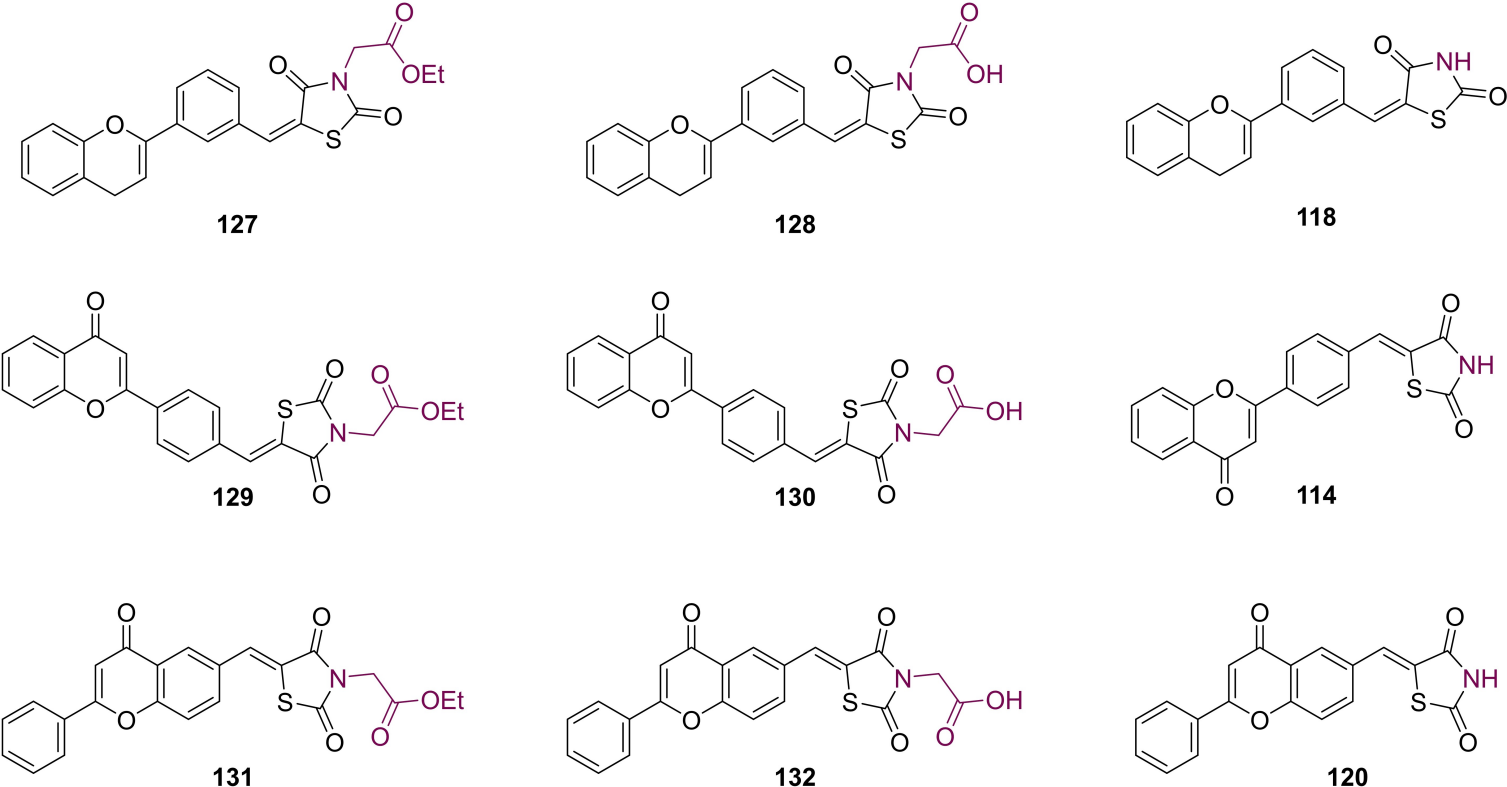
Flavone‐substituted derivatives featuring a benzylidene linker.

Functionalisation of the nitrogen to yield the acetate ester (**127**, **129**, **131**) proceeded by combining TZD (**3**) with ethyl bromoacetate and NaH in THF. Hydrolysis under acidic conditions then generated the free carboxylic acids (**128**, **130**, **132**). Coupling of TZD (**3**) with the appropriate flavone proceeded through KC, in the presence of NaOAc and glacial acetic acid, to yield the structures illustrated in Figure 17.^[105]^

*In vitro* ALR2 inhibition studies showed that the newly synthesised flavonyl compounds bearing the acetic acid chain (**128**, **130**, **132**) possessed high potency. In this study, ALR2 was isolated from kidney tissue obtained following the death of male albino rats and the formulated flavone‐substitued compound was dosed at a concentration of 100 μM. The highest potency was observed in the case of **128** which exerted an inhibitory action of 86.6 %. Compounds **130** and **132** were shown to inhibit ALR2 at 56.3 % and 44.6 % respectively at a concentration of 100 μM. The ester derivatives (**127**, **129**, **131**) generated however, proved to be less potent with percentage inhibition given as 12.9 %, 6.7 % and 14.4 % respectively at the same concentration. The decrease in potency was attributed to the lack of any acidic proton in the substrates. The presence of an acidic functionality is a highly important requirement for ALR2 inhibitors, because they form interactions in their ionised state.^[105]^

### 3.4 TZDs and Side‐effects

TZDs have received significant interest over the last three decades due to the risk of side‐effects. Such side‐effects were initally observed following the development of first generation glitazones as PPARγ inhibitors. Troglitazone induced liver damage has been attributed to to the production of harmful reactive metabolites during hepatic metabolism.^[107]^ This has been linked to acute liver failure caused by apoptosis of liver tissue cells.^[108]^ Further mechanisms of induced heptatoxicity include mitochondrial damage,^[109]^ promotion of oxidative stress and through the accumilation of bile in the liver due to inhibition of bile excretory proteins.^[109]^ However, it should be noted that specifically in the case of troglitazone, heptatoxicity is considered to be idiosynchratic and not dose‐ dependent.^[110]^

A second commonly witnessed side‐effect following prescripted use of TZDs is weight gain. TZDs are known to cause edema and increase the overal levels of plasma volume *in vivo*.^[111]^ This leads to a re‐distribution of fat *via* differentiation of preadipocytes into small fat cells.^[112]^

A more recently identified side‐effect following TZD usage concerns an increase risk in the development of bone fractures. Aside from weight gain being a contributory factor here,the main cause of this risk has been named ‘TZD‐induced bone loss’. Such induced action leads to an increase in adipogenesis and subsequent decrease in osteoblastogenesis.[^113^, ^114^] Furthermore, insulin levels play a direct role in the modulation of osteoblastogenesis and hence bone formation. As TZDs act to reduce insulin levels, the side‐effects of increased risk in bone fractions develop.^[115]^

## 4 Conclusion

The TZD core is a widely used structural motif within the sphere of medicinal chemistry. Its structure can be seen in a vast range of biologically active compounds in the treatment of many medical conditions. Its most common application can be seen in regards to the treatment of DM. DM, and specifically T2D, is considered to be one of the major risk factors associated with cardiovascular disease and mortality. TZD‐containing structures have been seen to inhibit a diverse range of biological targets not limited to PPARγ, PTP1B and ALR2. Unfortunately, TZDs have classically been associated with serious side‐effects. Such side‐effects commonly witnessed include severe hepatotoxicity, fluid retention and significant weight gain. As a result previously marketed glitazones including troglitazone and rosiglitazone were withdrawn from clinical use. The TZD structure has been the focus of many efforts to functionalise at two main positions, namely the activated methylene carbon (C_5_) and the amidic nitrogen (N_3_). Methodologies to substitute at these posistions are well reported and have stood up to scrutiny for several decades now. These ‘simple to replicate‘ methodologies offer synthetic organic and medicinal chemists the opportunity to develop a vast range of novel derivatives very quickly.

Following our recent publication in the late months of 2020 our group is currently developing a series of bioisosteric motifs containing TZD as a valuable tool for medicinal chemists.^[48]^

## Conflict of interest

The authors declare no conflict of interest.

## Biographical Information

*Nathan Long obtained his integrated masters degree at Queen Mary University of London (Pharmaceutical Chemistry) completing his final year research project under the supervision of Dr Stellios Arseniyadis on developing a photoinduced difluoromethylation methodology. Nathan has also worked in conjunction with the McCormack Group (William Harvery Research Institute) and Howell group (Queen Mary University of London) developing a series of positive allosteric modulators for the treatment of type 2 diabetes and arthritis. Nathan is currently working as a doctoral research student within the Wren group at Kingston University London currently developing a toolbox for the bioisosteric replacement for the carboxylic acid moiety*.

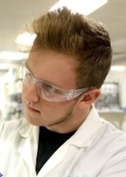

## Biographical Information

*Prof. Adam Le Gresley undertook his PhD at the University of Surrey under Prof Nikolai Kuhnert before completing his NIH postdoctoral research at Drexel College of Medicine, Philadelphia, USA. Since joining Kingston University as a lecturer in 2009, Adam has established a research group in organic and analytical chemistry, funded by industrial sponsors such as GlaxoSmithKline, LGC Ltd. and Innovate UK, working on the design and synthesis of fluorogenic compounds for problem pathogen detection and method development for NMR metabolomics/2D qNMR for complex mixture analysis and metrology. Adam was appointed full professor of organic chemistry in 2020*.

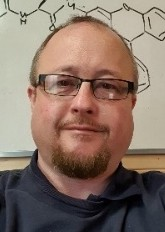

## Biographical Information

*Dr. Stephen Wren was educated at the Universities of Cambridge (PhD in Organic Chemistry, Corpus Christi College), Manchester and Texas (research in the synthesis of anti‐cancer compounds with Professor Phil Magnus at the University of Texas at Austin). Stephen is highly experienced in medicinal chemistry and has worked on a diverse set of biological targets over many disease areas in several organisations (Xenova, Argenta Discovery and Summit plc). He has an extensive track record in project and team management, intellectual property, drug discovery in many therapeutic areas. After three years at the Oxford Drug Discovery Institute and a Fellowship at St Hilda's College, Oxford, Stephen joined Kingston University London as a lecturer in organic and pharmaceutical chemistry in September 2018 and was promoted to senior lecturer in 2020*.